# Cancer drivers and clonal dynamics in acute lymphoblastic leukaemia subtypes

**DOI:** 10.1038/s41408-021-00570-9

**Published:** 2021-11-09

**Authors:** James B. Studd, Alex J. Cornish, Phuc H. Hoang, Philip Law, Ben Kinnersley, Richard Houlston

**Affiliations:** 1 Division of Genetics and Epidemiology, The Institute of Cancer Research, Sutton, SM2 5NG UK; 2 Present Address: Division of Cancer Epidemiology and Genetics, National Cancer Institute, Bethesda, ML USA

**Keywords:** Acute lymphocytic leukaemia, Cancer genetics

## Abstract

To obtain a comprehensive picture of composite genetic driver events and clonal dynamics in subtypes of paediatric acute lymphoblastic leukaemia (ALL) we analysed tumour-normal whole genome sequencing and expression data from 361 newly diagnosed patients. We report the identification of both structural drivers, as well as recurrent non-coding variation in promoters. Additionally we found the transcriptional profile of histone gene cluster 1 and *CTCF* altered tumours shared hallmarks of hyperdiploid ALL suggesting a ‘hyperdiploid like’ subtype. ALL subtypes are driven by distinct mutational processes with AID mutagenesis being confined to *ETV6-RUNX1* tumours. Subclonality is a ubiquitous feature of ALL, consistent with Darwinian evolution driving selection and expansion of tumours. Driver mutations in B-cell developmental genes (*IKZF1, PAX5, ZEB2*) tend to be clonal and RAS/RTK mutations subclonal. In addition to identifying new avenues for therapeutic exploitation, this analysis highlights that targeted therapies should take into account composite mutational profile and clonality.

## Introduction

Acute lymphoblastic leukaemia (ALL) is the most common childhood cancer, with around 80% of ALL cases derived from B-cell precursors (BCP-ALL) [1]. The disease is characterised by initiating genetic lesions resulting in characteristic patterns of chromosomal gain (hyperdiploidy), loss (hypodiploidy) or the formation of fusion genes. Recurrent fusions include t(12;21) *ETV6-RUNX1*, t(1;19) *TCF3-PBX1* and t(9;22) *BCR-ABL1* [1]. Copy number changes in *RUNX1*, caused by intra-chromosomal amplification (iAMP21), and *ERG* deletion, have also more recently been recognised as initiating events [2, 3]. The biological differences between subtypes is reflected in their clinical behaviour [4–7]. For example patients with hyperdiploid ALL have 5-year survival rate (5YSR) of > 90% [7]. In comparison around 60% of iAMP21 positive ALL will relapse resulting in 5YSR of only 29% [5].

Current first-line therapy for ALL is dominated by chemotherapeutic and steroidal agents. While their use has driven 5YSR to >90% [8], this is at the expense of significant morbidity, and despite these improvements, survival for relapsed ALL is only 21–39% [9, 10]. Strategies for developing novel therapies for ALL have largely focused on monoclonal antibodies or CAR-T cells. Such therapies are expensive; when licensed, the anti-cd19 monoclonal blinatumomab, was the most expensive cancer therapy ever [11]. It is therefore desirable to develop additional targeted small molecule therapies to reduce treatment-associated morbidity and relapse-associated cost. Such developments are likely to require precise molecular characterisation and risk stratification, informed by our understanding of ALL genomics.

Precancerous lesions harbouring initiating events can be undetected for years usually requiring the acquisition of additional genetic lesions for symptomatic disease. Common secondary lesions impact genes regulating the cell cycle (*CDKN2A*, *RB1*), B-cell development (*PAX5*, *IKZF1, EBF1*) and the RAS/RTK pathway (*NRAS, KRAS, FTL1*) [1]. Deletions of both *CDKN2A* and *PAX5* occur in around 30% of tumours. Activation of RAS-RTK genes while most common in hyperdiploid ALL, is observed in 35% of all tumours [12]. While the landscape of coding mutations in BCP-ALL has been well characterised [13–17], the full complement of molecular lesions sufficient to cause ALL, and explain its biological diversity are unknown.

To obtain a more comprehensive picture of the composite genetic events acting in concert in each of the BCP-ALL subtypes, we performed a genomic analysis of diagnostic samples from 361 ALL patients (Supplementary Fig. 1). We identify noncoding and copy number drivers. Our analysis also reveals differences in the mutational and biological pathways influencing the initiation and progression of disease subtypes.

## Methods

### Cases, data and sequencing

Matched tumour-normal whole genome sequencing (WGS) data from 361 treatment-naïve cases of paediatric (<18 years old) BCP-ALL were obtained from St. Jude Research Hospital (https://www.stjude.cloud/). Data were accessed and analysed through the DNAnexus cloud computing platform. Ethical permission was not required as all data were in the public domain.

WGS data were generated using 100 bp paired-end libraries (average read depth 45× and 62× for normal and tumour samples respectively), using Illumina (San Diego, USA) HiSeq2000 technology. Raw sequencing data were aligned with BWAmem v0.7.17 [18] to GRCh38, by Google Genomics. Cross-contamination was assessed using GATK v4.0.0.1; no sample having >2.6%. Tumour RNA sequencing (RNA-seq) on 222 (post quality control [QC]) of the 361 cases was performed on 125 bp paired-end libraries using Illumina HiSeq technology to an average number of 55 × 10^6^ reads. RNAseq fastqs were analysed using FastQC and aligned to GRCh38 using STAR v2.6.1 [19], discarding samples with <20% of reads aligning to the genome. Transcript abundance calculated in transcripts per million (TPM) using RSEM v1.3.0 [20] using GENCODEv30 annotation and was adjusted for batch effects using ComBat-seq [21].

Fusion genes were identified from RNAseq data using STAR-Fusion v1.5.018 and FusionInspector [22]. Candidate fusions were retained when fusion genes were separated by > 1MB and absent from control samples. For controls, we used unmatched lymphoblastoid cell line data (GTEx 1000 Genomes RNA sequencing project [23] [*n* = 465]) and unmatched mixed tissue samples (Human Protein Atlas Project [24] [*n* = 200]), processed using the same pipeline as tumour samples. Links provided in the web resources section.

The transcriptional impact of histone gene cluster 1 (chr6:26122685-26239852) deletion and *CTCF* alteration (deletion or mutation) were assessed using DESeq2 v1.329 [25], with default settings. Tumours were divided into three groups; two test groups possessing an alteration in *CTCF* or the histone gene cluster 1, excluding those with alterations in both and a control group. Test groups comprised those with gene(s) deletion (<2 copies), of which 17 tumours had associated RNAseq data (*CTCF* altered *n* = 9; histone 1 cluster altered *n* = 8).

### Variant calling

Somatic single nucleotide variants (SNVs) and indels were called in the 361 matched tumour-normal pairs using Strelka v2.8.4 [26] in tumour mode, adopting default parameters. QC filtering of somatic variants comprised: (1) Retaining only variants marked as ‘PASS’; (2) Excluding variants seen in the panel of 160 matched germline samples (generated by running Strelka2 in germline mode); (3) Excluding variants in repetitive regions (extracted from UCSC) or in homopolymer runs of >7 nucleotides; (4) Excluding variants with a POPMAX allele frequency >0.001 in GnomAD v3, (5) Excluding variants with a VAF < 5%. Driver mutation plot generated using Maftools [27].

### Mutational signatures

*De novo* extraction of signatures was performed using SigProfilerExtractor v1.0.18 [28]. Extracted signatures were assigned to reference signatures from Catalogue of Somatic Mutations in Cancer (COSMIC) v3.1 using a minimum cosine similarity threshold of 0.9.

### Identification of cancer drivers and pathways

Identification of SNV/indel drivers in coding regions was based on a consensus-based approach. Per gene *P* values were calculated combining the output of MutSigCV2 v3.11 [29], dndsCV v0.0.1 [30] and OncodriveFML [31] using Harmonic means [32] and Benjamini–Hochberg correction. Variants were classified as nonsilent using variant effect annotator (VEP) [33] annotations (Supplementary Table 1). Candidate drivers were filtered retaining genes: (1) significant (*P* < 0.05) in ≥2 methods, (2) corresponding RNAseq expression (mean > 0.02 TPM) and (3) mutated in ≥5 tumours.

To identify driver mutations in enhancer regions we adopted the strategy of Orlando et al. [34]. *Cis*-regulatory elements (CREs) were identified from promoter capture chromatin confirmation (PHi-C) contacts (CHiCAGO score > 5) from naïve B-cells [35]. CRE-specific mutation probabilities, for each tumour, were generated using logistic regression preformed with the *R* package glm, accounting for base composition, mutation rate, replication timing, and coverage. Replication timing was extracted for the lymphoblastoid cell lines (GM12878, GM12813, GM12812, GM12801, GM06990), link provided in the web resources section. The Poibin *R* package was used for approximation of Poisson binomials, deriving empiric *P* values regions as *per* Melton et al. [36].

CREs harbouring >5 mutations were examined for mutational clustering by permutation (*n* = 10,000) assuming uniform mutation distribution, deriving empiric *P* values. Frequency and clustering *P* values were combined using Fisher’s method and adjusted for multiple testing using Benjamini–Hochberg correction. Genome regions with a *Q* value < 0.1 were examined for transcriptional effects. Expression of genes were captured by an interaction were compared between mutated and non-mutated samples using Benjamini–Hochberg corrected Wilcox rank-sum test, excluding tumours with CNVs at either the target gene or CRE.

Mutation burden in promoters and UTR regions was assessed using OncodriveFML [31]. Promoters (defined from the transcription start site -2KB) were extracted from GENCODEv30 GRCh38.p12. Where genes had multiple transcription start sites all promoter sequences were evaluated jointly. Promoters were filtered for any overlapping coding or UTR sequence.

For pathway analysis driver genes were manually assigned to biological pathways. Gene—pathway assignments are described in Supplementary Table 2. To calculate the overrepresentation of alterations in biological pathways we compared the alteration frequency of each subtype to the remaining subtypes.

### Identification of copy number and structural variants

Somatic copy number variation (CNV) was called using CNVkit v0.9.5.3 [37]. Tumour WGS data were called against a pooled reference, generated from 45 representative matched germline samples (23 male, 22 female). CNVkit segment specific coverage log2 ratios were adjusted for tumour cell purity, estimated by cpgBattenberg [38]. Copy number states were assigned using default log2 thresholds (< −1.1 = 0, > −1.1 = 1, > 0.4 = 2, > 0.3 = 3, > 0.7 = 4). CNVs were considered ‘arm’ level when an alteration occupied > 80% of mappable chromosome arm length. Other variants were defined as ‘focal’. Additional copy number assessments were made using HMMcopy v1.32 calculating GC and mappability normalised tumour/normal log2 coverage ratios.

Driver CNVs were called using GISTIC2 v2.0.23 [39] run in focal mode (excluding arm level events) with default parameters. Genome regions were excluded if they: (1) overlapped a immunoglobulin locus, (2) contained no protein coding genes, (3) contained no genes expressed in corresponding RNAseq data (excluding deleted cases), (4) the region was significantly amplified and deleted, (5) *Q*-value > 0.01.

Structural variants (SVs) were called using Manta v1.5 [40], Lumpy v0.2.13 [41] and Delly2 v0.8.1 [42]. Manta and Delly2 were run using default parameters. Lumpy was run using the wrapper Smoove v0.2.3. Variants were excluded if they were located in centromeric, telomeric or heterochromatic regions, had a variant allele frequency (VAF) < 0.1, or occurred in a panel of matched normals, generated using the corresponding method. The remaining variants were merged as per Li et al. [43], retaining only those called by ≥2 methods.

SV cancer cell fractions (CCFs) were estimated using SVclone [44] and SV clustering examined using ClusterSV [43]. Regions of chromothripsis were identified using ShatterSeek v0.4 [45], based on thresholds of >3 adjacent segments of oscillating copy number involving >5 interleaved SVs. Candidate chromothripsis events were manually reviewed.

To jointly analyse CNVs and SVs, regions called by GISTIC were additionally filtered, retaining those enriched in overlapping SVs. For CNV regions, corresponding (deleted/amplified) simple SVs (not part of complex rearrangements called by SVClust) were examined. Chromosome-arm-specific background SV rates were estimated by permutation (*n* = 1000). *P* values were computed as the proportion of permutations where the number of simulated SVs overlapping a locus was greater than or equal to the number of observed SVs. Regions enriched (*P* < 0.01) in overlapping SVs were retained.

SV breakpoint motif enrichment was performed using HOMER v4.10.4 [46], by extracting two 100 bp sequences (±50 bp) from each breakpoint, excluding SVs where both breakpoints mapped to immunoglobulin regions (Supplementary Table 3). HOMER annotates motifs with the most similar sequence, based on Pearson *r*^2^, from the JASPAR [47] database. Annotated HOMER motifs were further processed with reference to motifs of candidate mutagenic drivers of ALL (Supplementary Table 4). Where the Pearson correlation between a HOMER motif and a candidate mutagenic motif exceeded the most similar JASPAR annotation they were substituted. Motifs with a correlation of < 0.85 were excluded.

### Tumour subtyping

Tumour chromosomal ploidy was based on copy number data from CNVkit. Tumours with a chromosome number > 50 were classified as hyperdiploid and those with < 45, hypodiploid. Near haploid tumours (n = 11) chromosome number 24–30 are included the hypodiploid subtype unless otherwise stated. iAMP21 status was called as *per* Harrison et al. [48]. Chromosomal ploidy was also called using clonal copy number calls from cpgBattenberg (*n* = 280). For samples with divergent chromosome numbers a manual review was performed, resulting in 3 samples being reclassified from hyperdiploid to near haploid.

Subtypes defined by fusion events (e.g., *ETV6-RUNX1*) were assigned based on clonal SVs consistent with fusion gene expression and corresponding fusion RNAseq expression. Cases without an established initiating driver event were designated as unclassified/other. The subtype composition of the cohort is detailed in Supplementary Fig. 2.

### Clonality and tumour evolution

Tumour ploidy and SNV cancer cell fractions (CCF) were estimated using cpgBattenberg v3.5.0 [38], adopting default parameters except minimum ploidy was thresholded at 1.1. Single nucleotide polymorphisms alleles from the 1000 Genomes Project (v3, GRCh38) were counted in tumour and normal samples, and genotypes phased using impute2 [49]. Purity-corrected copy number segments were used to compute SNV/indel CCFs and subclones identified by DPClust v2.28 [38], assigning somatic variants to clusters. Clusters with the highest CCF > 0.9 and < 1.1 were considered clonal. Samples were excluded based on the following criteria: (1) a variant cluster with CCF > 1.1; (2) no clonal cluster (CCF 0.9-1.1); (3) copy number state-specific SNVs which failed to cluster at predicted VAFs; (4) copy number solutions with homozygous deletions > 3 Mb. In the first instance, samples were analysed using Battenberg derived purity estimates, when resulting copy number solutions failed QC CCube v1.0 [50] estimates were used. 280 samples satisfying QC criteria were retained. Tumour cell purity estimates are detailed in Supplementary Fig. 3. To calculate driver gene mutation burden in clonal and subclonal compartments we used cluster assignments from DPClust. The frequency of clusters containing ≥1 damaging driver (Supplementary Table 5) mutations were calculated for each subtype and compared to the remaining subtypes. Heterogeneity was estimated using the Simpson Index (probability that two individuals/cells, selected from a population/tumour, are from the same species/clone), calculated using VEGAN [51]. Evidence to support neutral evolution was sought using MOBSTER v0.1.1 [52], as per authors recommendations (retaining only SNVs and indels in diploid regions). MOBSTER identifies variants with a VAF distribution consistent with neutral evolutionary processes, termed a “neutral tail”. Variants belonging to neutral, subclonal or clonal clusters were analysed using dNdSCV. The mutation rate of ALL drivers in neutral, subclonal or clonal clusters was calculated as the number of nonsynonymous variants/all non-synonymous sites/total number of mutations (Supplementary Table 5).

## Results

### Mutation burden

**Fig. 1:**
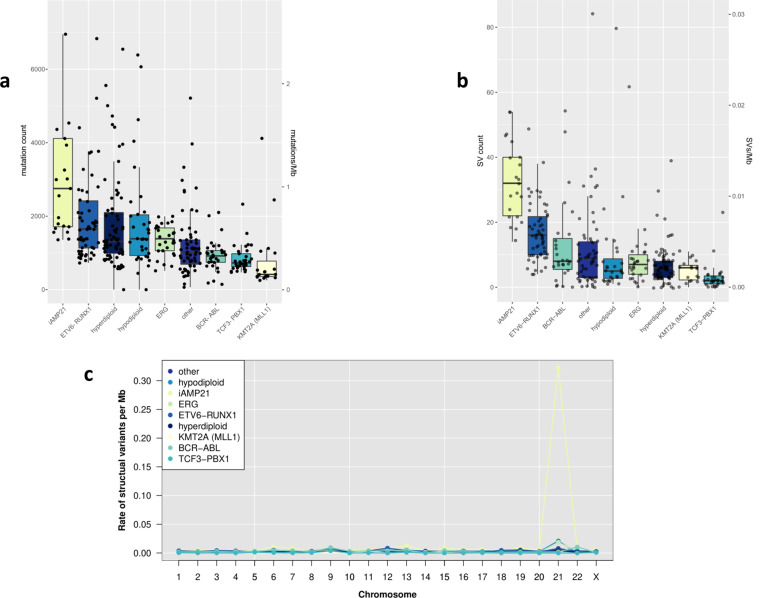
Mutation burden by subtype. Short somatic variants (SNVs and indels) were called in 361 matched normal/tumour whole genome sequencing samples. **a** Burden of SNVs and indels. Box and whiskers plot of mutation count per tumour. **b** Burden of structural variants (SVs). Box and whiskers plot of SV count, dots represent individual tumours. **c** Plot of the SV rate per chromosome retaining only intrachromsomal variants outside immunoglobulin loci. *Y-*axis; mean SVs rate per Mb. *X-* axis; chromosome.

As previously documented, the burden of SNVs and indels was low (median 0.43 Mb^-1^, range 0.023-5.29) when compared to the majority of solid cancers. Mutation burdens differed significantly across subtypes (*P*_Kruskal-Wallis_ = 2.2 × 10^−16^), iAMP21 and KMT2A (MLL1) positive tumours having the highest and lowest burdens respectively (Fig. 1a). The most common chromosome-arm level aberrations were loss of 9p (containing *CDKN2A*/*CDKN2B*) and gain of 21q (containing *RUNX1*), both occurring in 8% of cases (Supplementary Fig. 4). 21q gain occurred preferentially in hypodiploid tumours (*Q* = 0.10), whereas 9p loss was most frequent in *TCF3-PBX1* translocated tumours (*Q* = 0.19), and (Supplementary Fig. 5a and 5b).

The median number of SVs was eight per tumour (2 × 10^−3^ Mb^−1^), iAMP21 tumours possessing the highest number (Fig. 1b). The genome-wide distribution of SVs is shown in Supplementary Fig. 6. Chromosome 21 SVs rates were 10-fold higher (0.027 Mb^−1^) than other chromosomes, largely accounted for by iAMP21 tumours (Fig. 1c). Since iAMP21 tumours are defined by *RUNX1* copy number, we examined the distribution of SVs on chromosome 21, finding no clustering evident (Supplementary Fig. 7). Chromothripsis did not account for elevated SV rates in iAMP21 tumours as no events were observed on chromosome 21.

### Identification of driver genes

We searched for drivers of ALL by first considering the following classes of somatic coding alterations; single nucleotide variants (SNVs)/indels, copy number variants (CNVs), structural variants (SVs) and loss of heterozygosity (LOH). In addition to established drivers, we identified a number of candidate novel ALL drivers, including *HLA-DRB5*, the histone gene cluster 1*, USP8* and *CHID1*.

**Fig. 2:**
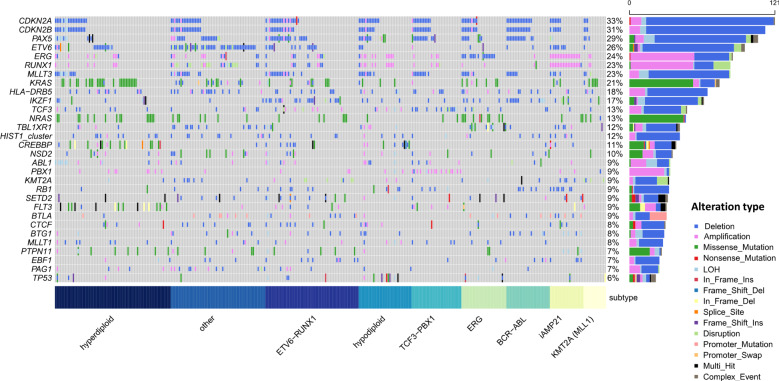
Driver gene analysis. Oncoplot of somatic alterations for selected ALL driver genes (Supplementary Table 10), genes altered in > 20 tumours, compiled from SNV, indel, CNV (only focal events), SV, RNAseq, and LOH. Vertical lines represent one tumour. Coloured sections in grey grid denote alteration type, described in key - “Alteration type”. Short nucleotide variants span the entire row, other alterations span half the row width. The right bar plot shows the frequency of driver gene alteration, colour denotes alteration type, as prior. Deletions and amplifications derived from CNVs and SVs; disruption from RNAseq and SVs. Alterations are shown non-redundantly; tumours with multiple alterations in the same gene only counted once. Plot generated using Maftools [6] after the exclusion of genes in the psuedoautosomal regions.

**Fig. 3:**
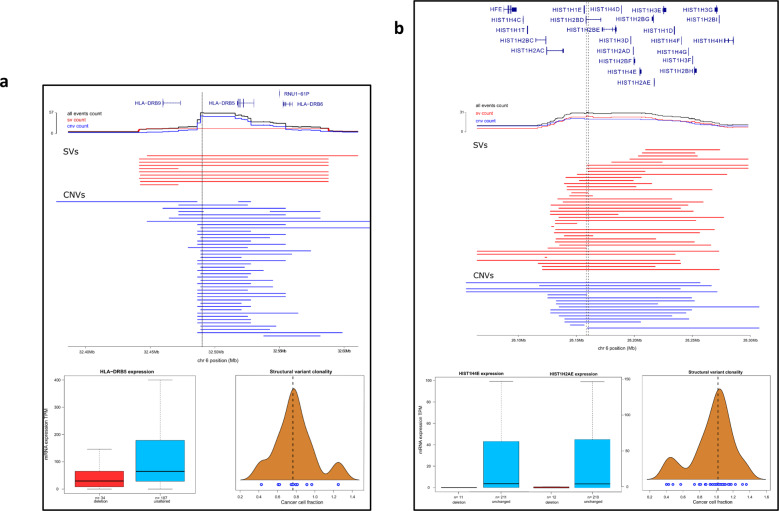
Recurrent copy number and structural variants. Significantly amplified or deleted regions in CNV data, were filtered retaining only those with an enrichment of structural variants, based on a permutation test. Regional genetic plots showing recurrent deletions mapping to (**a**) *HLA-DRB5* and (**b**) histone gene cluster 1. Upper panes shows gene position. Line plots show number of tumours with an overlapping variant; blue—CNVs, red—SVs, black—total count (tumours with both SVs and CNVs counted once). Central pane shows the individual variants. For convenience only variants starting or ending in the field of view are plotted. Vertical black lines denote region with the highest deletion frequency. Lower left pane, box plots of gene expression split by mutational status. Lower right pane, density plots of structural variant clonality; blue circles individual SVs. Genomic coordinates from GRCh38.

Consistent with previous reports [1, 53], the most frequently altered genes included *CDKN2A/B, PAX5, ETV6, ERG, RUNX1, NRAS, KRAS* and *IKZF1* (Fig. 2). By jointly analysing CNV and SV data, we identified two novel regions of recurrent alteration. Firstly, a 120 kb region of *HLA* (6p21; 32,442,465-32,554,750 bps) was deleted in 17% of tumours (Fig. 3a). Within this region only *HLA* − *DRB5* was expressed and deletion was associated with significantly reduced gene expression (*P*_Mann-Whitney_ = 3.7 × 10^−4^). We further evaluated read depth data in tumours with an HLA-DRB5 SVs but lacking a CNV using an additional copy segmentation algorithm [54], finding evidence of a corresponding change number change within 2,000 bp an SV breakpoints in every tumour (Supplementary Fig. 8). Secondly, 117 kb of 6p22.2 overlapping histone gene cluster 1 (26,122,685-26,239,852 bps) was deleted in 10% of tumours (Fig. 3b), deletion was associated with reduced expression of HIST1H4E (*P*_Mann–Whitney_ = 0.034) and HIST1H2AE (*P*_Mann–Whitney_ = 0.023). We estimated SV cancer cell fraction (CCF) using SVclone [44] finding the majority of these variants in the region are clonal.

We observed nonsilent SNVs or indels in *USP8*, *BSN* and *SLC35G5* in 1.9, 1.7 and 1.4% of tumours respectively (Supplementary Tables 6,7 and Supplementary Fig. 9). *USP8* missense mutations were clustered at three base positions, consistent with oncogenic activation (Supplementary Fig. 10). While the frequency of *USP8* variants in the gNOMAD v3 database was < 0.001, further curation revealed each variant occurred above frequency filter thresholds in legacy releases of the ExAC database indicating they may be technical artefacts. None of the variants in *BSN* were recurrent and all predicted to be damaging by SIFT and PolyPhen (Supplementary Table 7). Variants in *SLC35G5* were predicted to be benign and occurred in ExAC inconsistent with driver function.

**Fig. 4:**
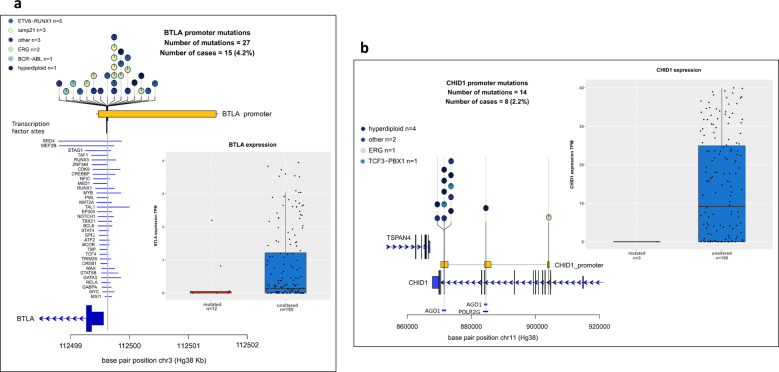
Non-coding driver mutations. Mutation burden within promoters and their transcriptional impact. Promoter mutations of (**a**) *BTLA* and (**b**) *CHID1*. Regional plot of mutations (coloured circles) relative to coding sequence (dark blue boxes) and promoter (yellow horizontal bar). Transcription factor binding sites (light blue horizontal line) overlapping mutations were extracted from Encode and ChIP atlas. Grey boxes correspond to transcriptional impact on respective gene. Box and whiskers plot, tumours are split by mutational status, dots represent individual tumours.

Next, we sought to identify non-coding driver mutations. We observed a significant excess of promoter mutations for *BTLA* (4.2%, *Q* = 0.002) and *CHID1* (2.2%, *Q* = 0.049). *BTLA* promoter mutations clustered within a 27 bp region, and were associated with 5-fold reduced BTLA expression (*P*_Mann–Whitney_ = 0.056), the small number of tumours with expression data presumably preventing this relationship attaining significance (Fig. 4a). By analysing transcription factors (TFs) with evidence of *BTLA* promoter binding in ChIPseq, we found each variant tumour possessed a mutation predicted to disrupt TF binding, most frequently RUNX1/3, GATA3 and MYB (Supplementary Table 8). Of 14 *CHID1* promoter mutations 12 clustered within a 12 bp region 1 kb upstream of the TSS and within an AGO1 binding site, corresponding RNAseq was consistent with variants reducing *CHID1* expression (Fig. 4b).

To search for significantly mutated *ci*s-regulatory elements (CREs) we restricted our analysis to sequences interacting with promoters through chromatin looping in naïve B-cells [35]. We observed no CREs possessing an excess of mutations and associated with the expression of interacting genes. Additionally we found no evidence of recurrent mutations within UTRs or noncoding RNAs, imposing a threshold of at least five affected tumours.

### Mutated pathways

We next assessed the subtype specificity of driver variation. Additional to documented enrichment of *NRAS*/*KRAS* mutations in hyperdiploid ALL and *TP53* mutations in hypodiploid/near haploid ALL, we identified a number of other associations (Supplementary Table 9). Notably, *TBL1XR1* and *ZEB2* mutations were enriched in *ERG*-deleted ALL (present in 21% and 14% of tumours, respectively). iAMP21 tumours were characterised by excess *RB1* deletion (40%) and *IL7R* mutation (20%). *NF1* mutations were largely confined to near haploid tumours occurring in 45%. Finally, *ETV6-RUNX1* positive tumours were associated with enrichment for *TBLXR1* and RAG1/RAG2 deletions.

Given the identification of deletions within the histone gene cluster 1 and the previous identification of *CTCF* as a potential ALL driver we explored the transcriptional impact of these lesions, analysing differential expression. We divided tumours according to *CTCF* (mutated/deleted) and histone 1 cluster (deleted) status, excluding tumours variant for both. We identified five differentially expressed genes in both sets of mutated tumours (*P*_Binomial_ = 1.5 × 10^−8^), including *CLIC5* and *IGF2BP1* (Supplementary Table 10 and Supplementary Fig. 11). *CLIC5* and *IGF2BP1* have been identified as markers of hyperdiploid ALL [55], however, none of the test tumours used in this analysis were hyperdiploid. In total 60 tumours (17%) harboured alterations (deletions or mutations) in either *CTCF* or the histone gene cluster 1.

**Fig. 5:**
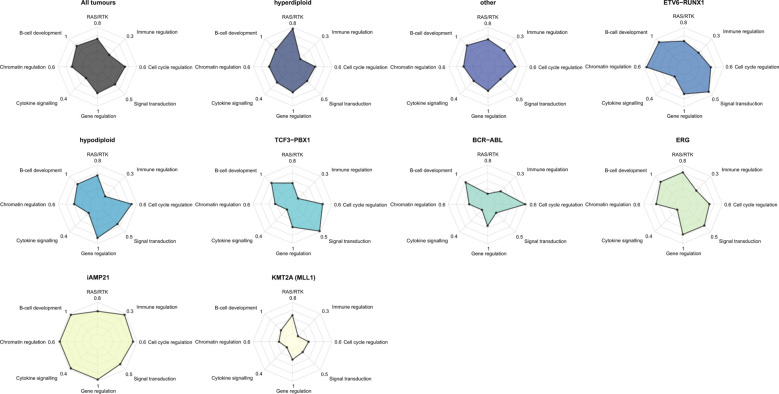
Pathway analysis and signature analysis. Radar plots showing the most frequently altered pathways for each subtype. Driver genes grouped according to the biological pathway. Somatic alterations for a selected ALL driver genes was compiled from SNV, indel, CNV, SV, RNAseq, and LOH data (CNVs include only focal events). Subtype defining events are excluded (e.g, disruption of *ETV6* or *RUNX1* in ETV6-RUNX1 positive tumours). The proportion of tumours with an alteration in any gene assigned to that pathway is plotted on the radial axis. Each axis is scaled separately. Gene—pathway assignments: RAS/RTK; *NRAS, KRAS, PTPN11, FLT3, NF1, ABL1*. B-cell development; *PAX5, IKZF1, ETV6, ZEB2, RUNX1, TCF3, RAG1, RAG2, EBF1*. Chromatin regulation; *SETD2, HDAC7, NSD2, CTCF, KMT2A, STAG2*, histone gene cluster 1. Cytokine signalling; *JAK2, IL7R, CRLF2*. Gene regulation; *CREBBP, MLLT1, MLLT3, AFF1, BTG1, ERG, TCF4, NCOA6*. Signal transduction; *TBL1XR1, TBL1X, PBX1, PAG1*. Cell cycle regulation; *CDKN2A, CDKN2B, RB1*. Immune regulation; *BTLA, HLA-DRB5*.

To produce a composite picture of somatic events we clustered drivers by biological pathways (Fig. 5, Supplementary Tables 2 and 11). Alterations of B-cell development genes, were the most frequent, found 70% of tumours. This analysis confirmed the importance of RAS/RTK alterations in hyperdiploid biology and highlighted a number of other key pathways. Secondary alterations affecting cytokine signalling occurred in 37% iAMP21 of tumours (*Q*_Binomial_ = 2.2 × 10^−3^) involving *IL7R*, *JAK2* or *CRLF2* (including 3/5 cases of *P2RY8-CRLF2* translocation). Alteration of chromatin regulating genes occurred in 56% of *ETV6-RUNX1* positive tumours (*Q*_Binomial_ = 1.9 × 10^−4^). Hypodiploid tumours were typified by disruption of transcriptional regulators (*Q*_Binomial_ = 0.012), while *TCF3-PBX1* tumours were overrepresented in disruption to genes regulating signal transduction (*Q*_Binomial_ = 0.012).

**Fig. 6:**
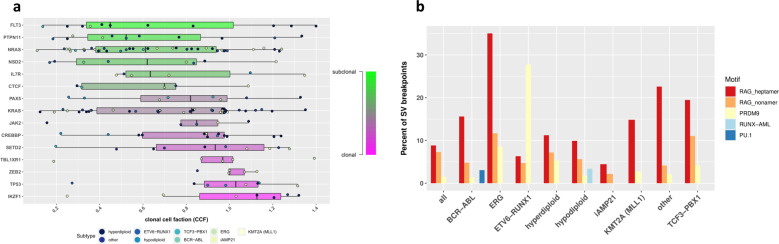
Driver clonality and SV breakpoint enrichment. **a** Driver gene mutation (SNV/indels) clonality. Box and whiskers plot showing the proportion clonal mutations for ALL driver genes. Each circle represents a mutation, coloured according to disease subtype, for tumours with multiple mutations in the same gene the variant with the highest clonal cell fraction is retained. **b** Structural variant motif enrichment. Bar chart showing motif enrichment at SV breakpoints. Two 100 bp of sequences flaking each breakpoint of an SV were extracted and analysed using HOMER. Y-axis; percent of extracted sequences containing motifs.

We assessed driver gene mutation clonality, finding most occur both the clonally and sub-clonally (Fig. 6a and Supplementary Fig. 12**)**. An exception was *ZEB2* where mutations were always clonal, moreover, mutations of B-cell development and haematopoiesis genes (*IKZF1*, *PAX5* and *ZEB2*) tended to be clonal. Conversely the majority of RAS/RTK gene mutations were subclonal (65%; *P*_Fisher_ = 0.001). This was especially true of ERG-deleted tumours where 44% possessed a subclonal RAS/RTK variant (accounting for 89% of RAS/RTK mutations in the subtype) compared to 8% with a clonal variant. Conversely RAS/RTK mutations in hyperdiploid tumours were usually clonal (60%), occurring in 44% of tumours compared to 20% with only a subclonal variant.

### Mutational signatures

To examine factors promoting tumorigenesis we extracted COSMIC single base signatures (SBS) using SigProfilerExtractor [28]. Ten signatures contributed >1% of mutations (Supplementary Fig. 13). SBS5 (aetiology unknown but clock-like) accounted for the most mutations (40%) and was seen in all tumours **(**Supplementary Figs. 14 and 15). SBS2 and SBS13 (AID/APOBEC) were almost exclusively confined to *ETV-RUNX1* tumours (*Q*_Man–Whitney_ = 2.3 × 10^−33^ and *Q*_Man–Whitney_ = 1.1 × 10^−36^ respectively), whilst SBS7a (UV exposure) was highly enriched in iAMP21 tumours (*Q*_Man–Whitney_ = 5.3 × 10^−12^) (Supplementary Figs. 16 and 17). SBS7a was associated with the highest mutation rate, 10-fold higher than SBS1 (Supplementary Fig. 18) and was largely responsible for the increased mutation rate in iAMP21 tumours (Supplementary Fig. 19).

SVs in *ETV6-RUNX1* positive tumours bear the hallmarks for RAG1 and RAG2 activity [56]. We searched for recurrent DNA motifs at SV breakpoints, firstly agnostically by motif enrichment using HOMER [46], and secondly by assessing the similarity of discovered motifs to the binding sites of candidate mutagenic drivers (Supplementary Table 4). Cohort wide, the most enriched sequences were the RAG heptamer (*P* < 1 × 10^−200^), RAG nonamer (*P* < 1 × 10^−200^) and PRDM9 binding motif (*P* = 1 × 10^−121^), found at 8.8, 7.2 and 1.5% of breakpoints respectively. With the exception of *ETV6-RUNX1* positive tumours the most frequent enriched motifs were the RAG heptamer and RAG nonamer, however in *ETV6-RUNX1* the most common was the PRDM9 binding motif contained in 28% of breakpoints (*P* = 1 × 10^−162^) (Fig. 6b). Overall RAG heptamers were observed in both breakpoints of 3% of SVs.

We also sought evidence of activation-induced deaminase (AID) activity at SV breakpoints. Due to the degenerate nature of AID motifs we used the number of repeats of core AID recognition sequences (Supplementary Table 4) as a proxy of activity. After comparing SVs in immunoglobulin regions we established a cut-off of > 10 repeats as indicative of AID activity (Supplementary Fig. 20). AID signatures were detected in the breakpoints of 2% of all SVs, but 17% of SVs in *TCF3-PBX1* positive tumours (*P*_Fisher_ = 8 × 10^−9^) (Supplementary Fig. 21).

### Clonal architecture

**Fig. 7:**
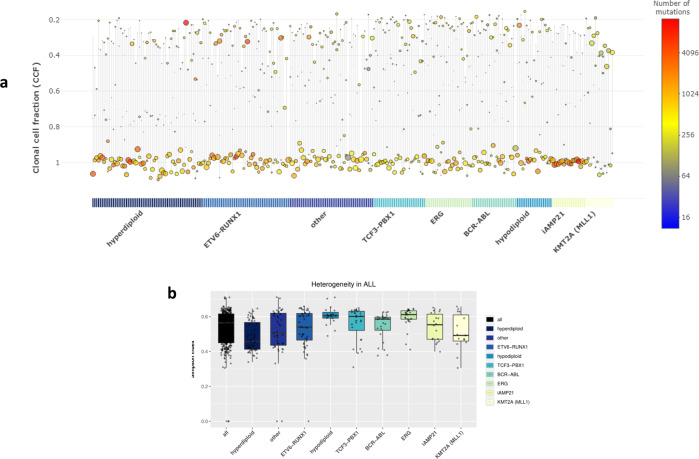
Clonal architecture and evolution. Variant cancer cell fractions (CCF) were calculated and variants clustered into clonal and subclonal populations. **a** Distribution of clones. Horizontal lines represent single tumours, circles represent clones; the size and colour of circles corresponding the proportion and number of variants assigned to each clone. Y-axis; clonal frequency (proportion of cell cells with a variant(s)). **b** Heterogeneity between subtypes. Box and whiskers plot of Simpson index (higher values indicative of increased heterogeneity). Each dot corresponds to a tumour.

The presence of subclonal populations in tumours was almost universal (observed in 98% of tumours; Fig. 7a). Most commonly tumours possessed two subclones, however, *ERG*-deleted tumours tended to have a higher number (*Q*_Mann–Whitney_ = 0.008) and KMT2A translocated lower (*Q*_Mann–Whitney_ = 0.038) (Supplementary Fig. 22). The distribution of subclone CCF was similar across subtypes, with the exception of hyperdiploid tumours whose subclones had higher CCFs (*Q*_Mann–Whitney_ = 0.004), 50% having a subclone with a CCF between 0.7 and 0.8, compared to 9% of other tumours (Supplementary Fig. 23).

The diversity of cell populations (*i.e*. heterogeneity) varied across subtypes, hypodiploid and *ERG*-deleted tumours were the most heterogeneous (median Simpson index = 0.61 and 0.62; *Q*_Mann-Whitney_ = 1.7 × 10^−3^, 1.13 × 10^−3^), while hyperdiploid tumours exhibited lower heterogeneity (Simpson index = 0.45; *Q*_Mann–Whitney_ = 2.8 × 10^−7^) (Fig. 7b).

Accounting for mutational frequency, subclones were enriched in driver mutations (*P*_Binomal_ = 1.8 × 10^−5^) relative to clonal populations. To examine the processes influencing tumour evolution we enumerated the number of subclones with (≥1) driver gene mutations for each subtype, comparing this to the frequency observed in remaining subtypes. *ERG*-deleted tumour subclones were most likely to possess drivers mutations (35%; *Q*_binomal_ = 0.0016), whereas *BCR-ABL1* positive tumour subclones contained the lowest frequency (4%; *Q*_Binomal_ = 0.052) (Supplementary Fig. 24).

To explore the possible contribution of neutral evolution to tumour heterogeneity we used MOBSTER [52], which models variant distribution under neutral evolutionary processes. MOBSTER called neutral ‘tails’ in the majority of tumours, fitting a median of 12% (SNVs) and 16% (SNVs and indels) of variants (Supplementary Fig. 25). Using dNdSCV we found evidence of positive selection in neutral tail compartments which were enriched in *NRAS* (*Q* = 3.4 × 10^−8^) and *KRAS* (*Q* = 1.9 × 10^−3^) mutations. Additionally, rates of non-synonymous substitution in *NRAS*, *KRAS*, *FLT3, NSD2* were higher in tail compartments than clonal compartments (Supplementary Fig. 26).

## Discussion

By analysing whole genome sequencing and transcriptome data from a large series of ALL patients, we provide for an enhanced understanding of ALL subtype genetics identifying novel candidate coding, noncoding and copy number drivers. Our analysis reveals differences in the mutational and biological pathways processes influencing the initiation and progression of the disease. We also provide evidence of the ongoing selection of subclonal mutations as a ubiquitous feature of ALL evolution.

Around half of *ETV6-RUNX1* and iAMP21 tumours are characterised both by an increased mutation rate and enrichment for specific COSMIC single base signatures. AID/APOBEC related signatures, SBS2 and SBS13, were confined to *ETV6-RUNX1* ALL, while UV-associated SBS7a was highly enriched in iAMP21 positive tumours. SBS7a has previously been reported to occur in ALL tumours at a similar rate [28]. Moreover, SBS7a occurs at similar rates in a number of tumours types lacking UV exposure [28]. These observations provide evidence for an additional mechanistic basis for SBS7a thus implicating unknown germline genetic or environmental factors promoting tumourigenesis of iAMP21 tumours.

We identified three genes enriched in short nucleotide variants, *USP8*, *BSN* and *SLC35G5*. The presence of known sequencing artefacts in *USP8* and *SLC35G5* necessitates further validation to establish the candidacy of these genes as drivers. *BSN* is predominantly expressed in neurons where it regulates the release of neurotransmitters consistent with these findings being coincidental.

Deletions of the gene encoding B and T-lymphocyte attenuator (*BTLA*) have previously been reported in ALL [57], which typically overlap *CD200*, however, the specific functional mediator has yet to be elucidated. The existence of BTLA promoter SNVs are consistent with this gene, as opposed to CD200, being the driver gene at the 3q12.2 region.

We additionally report recurrent copy and structural variation impacting *HLA-DRB5*. Although the selective basis of these lesions remains unclear, genome-wide association studies in chronic lymphocytic leukaemia [58] and lymphoma [59] have identified germline variants in *HLA-DRB5* influencing disease risk.

Around 10% of tumours possessed a deletion overlapping the histone gene cluster 1, which contains 16 different histone isoforms, including at least two of each core histone. Recurrent histone H1 mutations have also been reported in around 30%-50% of lymphomas altering chromatin architecture and inducing stem cell-like transcriptional profiles [60]. The further functional characterisation will be required in order to determine the functional gene(s) within these lesions. We show that tumours harbouring histone gene cluster 1 deletions and *CTCF* alterations share a common transcriptional profile, both down-regulating *IGF2BP1* and *CLIC5*. Interestingly, these genes were recently identified alongside *CTCF* as markers hyperdiploid tumours [55]. No hyperdiploid tumours were included in this analysis, precluding a co-variant effect driving this relationship. Mutations in these genes were however enriched in tumours with no assigned subtype. Alteration of either *CTCF* or histone gene cluster 1 was common, occurring in 17% of tumours. Collectively these data raise the possibility of a ‘hyperdiploid-like’ subtype of ALL.

While there is commonality in disruption of pathways between ALL subtypes there are clear distinctions, not only in the particular biological pathways harbouring mutations but also the clonal distribution of these mutations. These differences have implications for choice of potential targeted therapies and determining which patients will benefit most from their use. As targeting activated oncogenes is generally more tractable than tumour suppressors the biological pathways of most relevance for ALL are RAS/RTK and IL7 signalling. Importantly, RAS/RTK mutations in hyperdiploid tumours were typically clonal, whereas in *ERG*-deleted ALL mutations were almost exclusively subclonal, suggesting the efficacy of RAS/RTK inhibitors will differ between subtypes. Alteration of IL7 signalling was common in iAMP21 tumours suggesting that JAK2 inhibitors may have utility in this group.

Somatic variants identified as neutrally occurring by MOBSTER were enriched in ALL drivers, indicating that neutral evolution is not a major contributor to genetic heterogeneity in ALL, this may be reflective of the low mutation rate of the disease comparative to most solid cancers. We show that subclonality in ALL is common suggesting Darwinian evolution drives the selection and expansion of mutations and subclones. Consequently, the use of novel targeted therapies should take account of the clonality and heterogeneity of tumours.

### Web resources

Repetitive genomic loci used for variant filtering were downloaded from hgdownload.cse.ucsc.edu/goldenpath/hg38/database/simpleRepeat.txt.gz.

Replication timing was downloaded from 2.replicationdomain.com/.

Smooth the wrapper for structural variant caller Lumpy is available from github.com/brentp/smoove.

Structural variant positional filtering was based on https://github.com/dellytools/delly/blob/master/excludeTemplates/human.hg38.excl.tsv.

FusionInspector the RNAseq fusion gene detection software is available from github.com/FusionInspector.

Control RNAseq data for GETex and HPA were downloaded from ebi.ac.uk/arrayexpress/experiments/E-GEUV-1/samples/ and ftp://ftp.sra.ebi.ac.uk/vol1/run/ERR315.

Promoters were defined using genode v30 downloaded from ftp://ftp.ebi.ac.uk/pub/databases /gencode/Gencode_human/release_30/ gencode.v30.annotation.gtf.gz.

VEGAN package for calculating population diversity is available from github.com/vegandevs/vegan.

HMMcopy is available from http://www.bioconductor.org/packages/release/bioc/manuals/HMMcopy/man/HMMcopy.pdf

## Supplementary information

Supplementary figures

supplementary tables

SUPPLEMENTARY FIGURE and TABLE LEGENDS

## Data Availability

Data available from DNAnexus (DNAnexus.com) subject to application from St Jude hospital.

## Code availability

Any unpublished/additional code is available on request from the author.
