# Supplementary material for: Cancer drivers and clonal dynamics in acute lymphoblastic leukaemia subtypes
Source: Blood Cancer J. 2021 Nov 9;11(11):177. doi: 10.1038/s41408-021-00570-9 (PMC8578656; doi:10.1038/s41408-021-00570-9)

Supplementary Figures

Supplementary Fig. 1

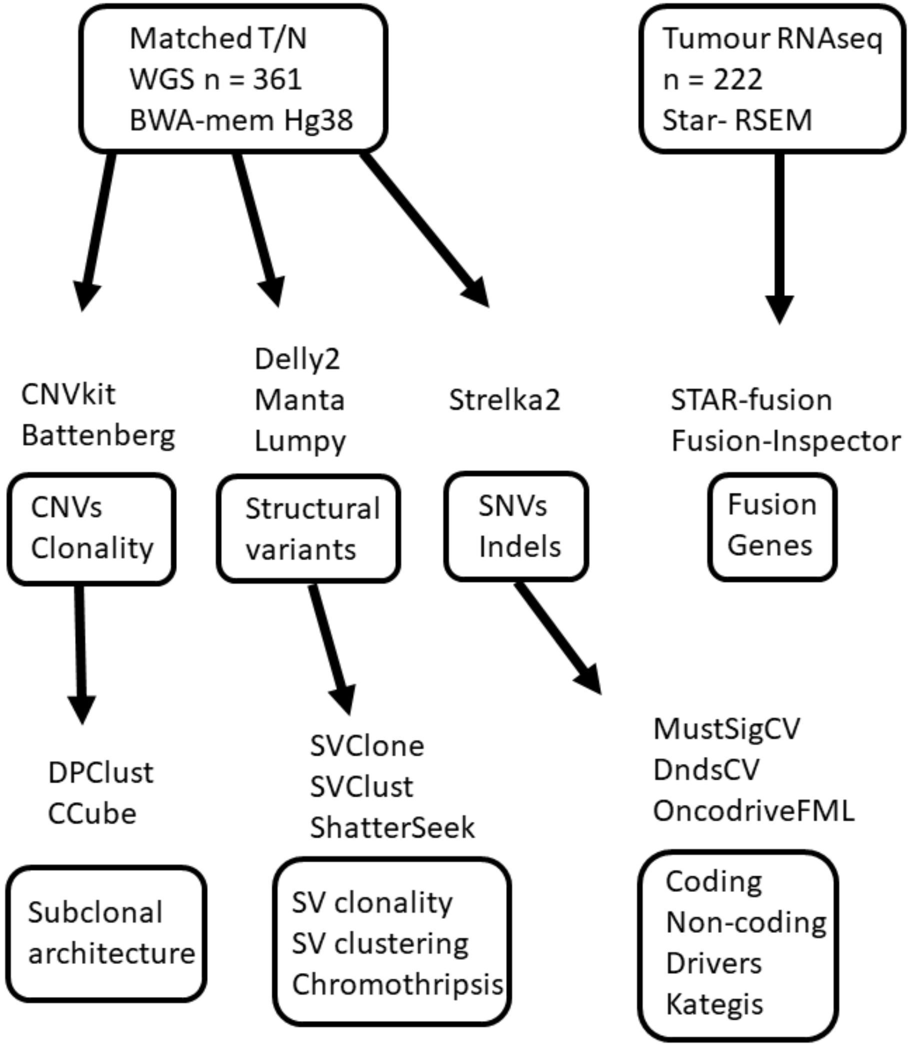

Supplementary Fig. 2

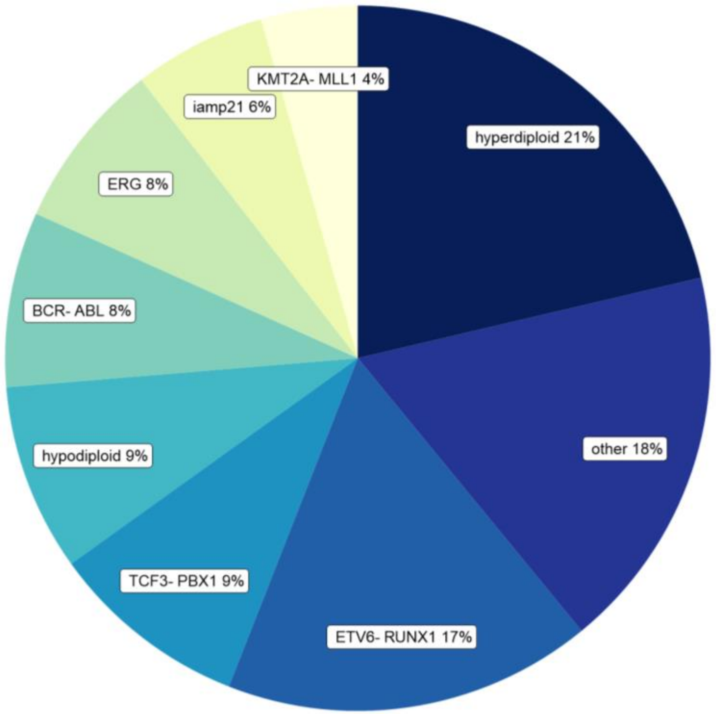

Supplementary Fig. 3

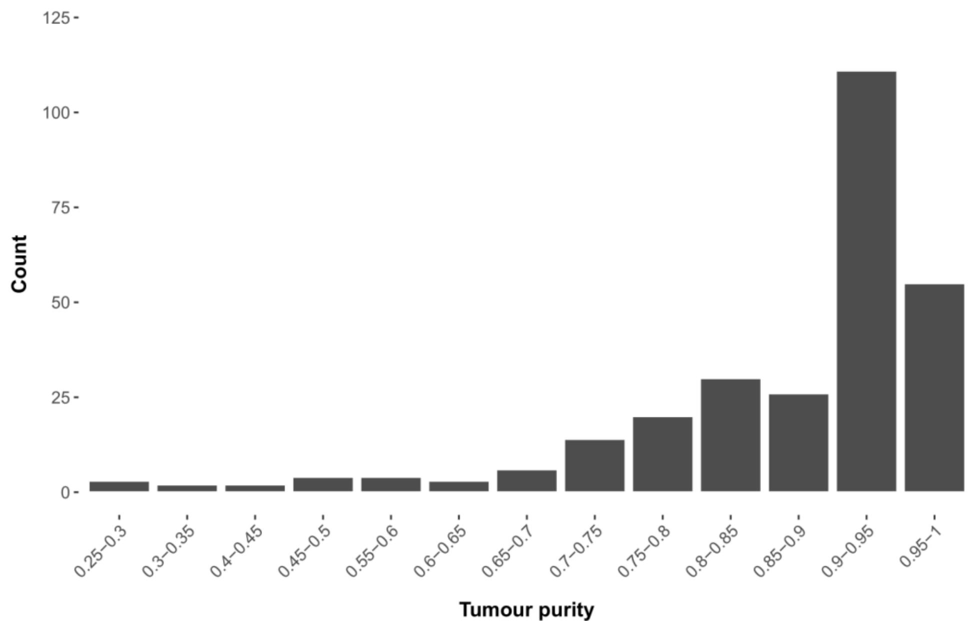

Supplementary Fig. 4

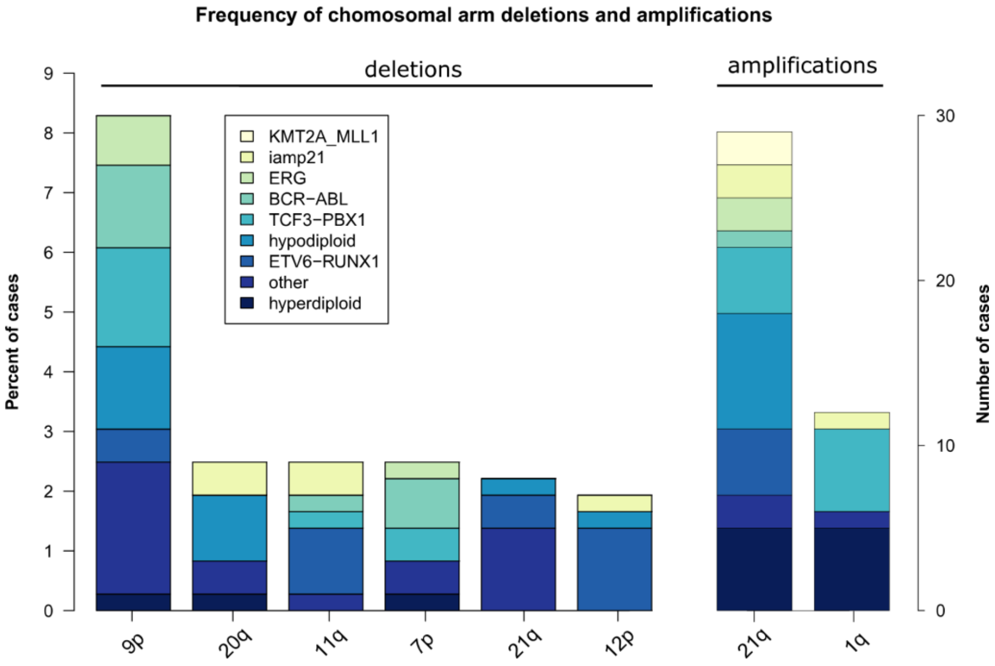

Supplementary Fig. 5

a

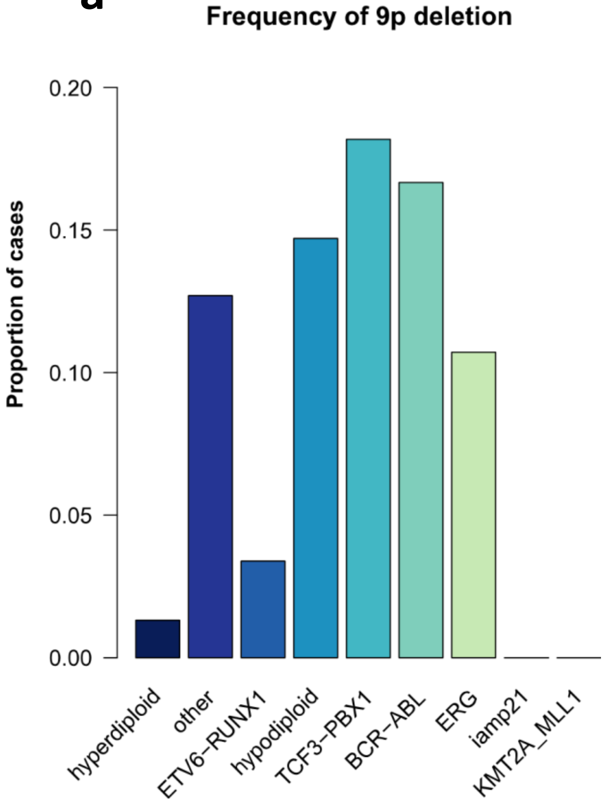

b

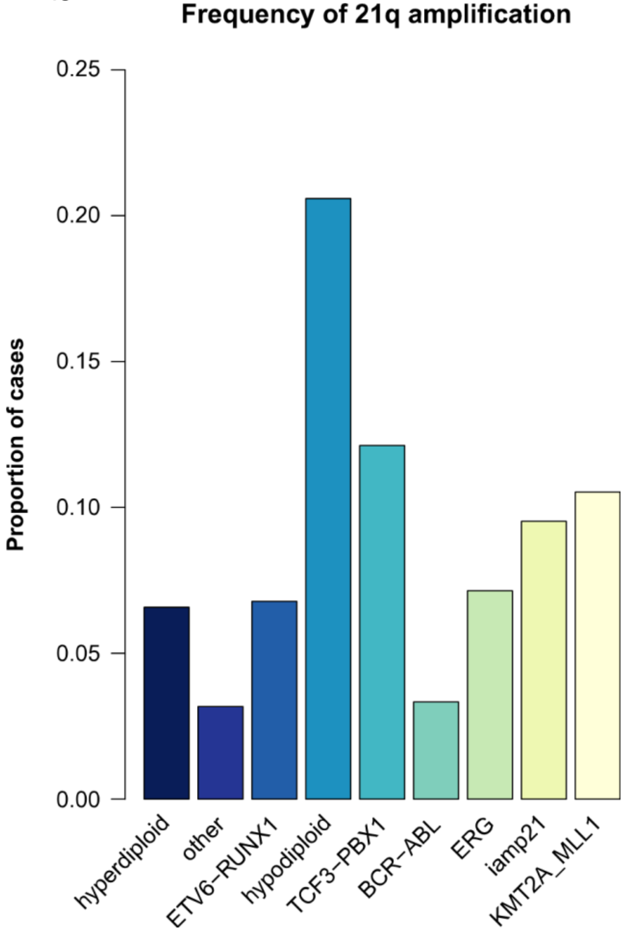

Supplementary Fig. 6

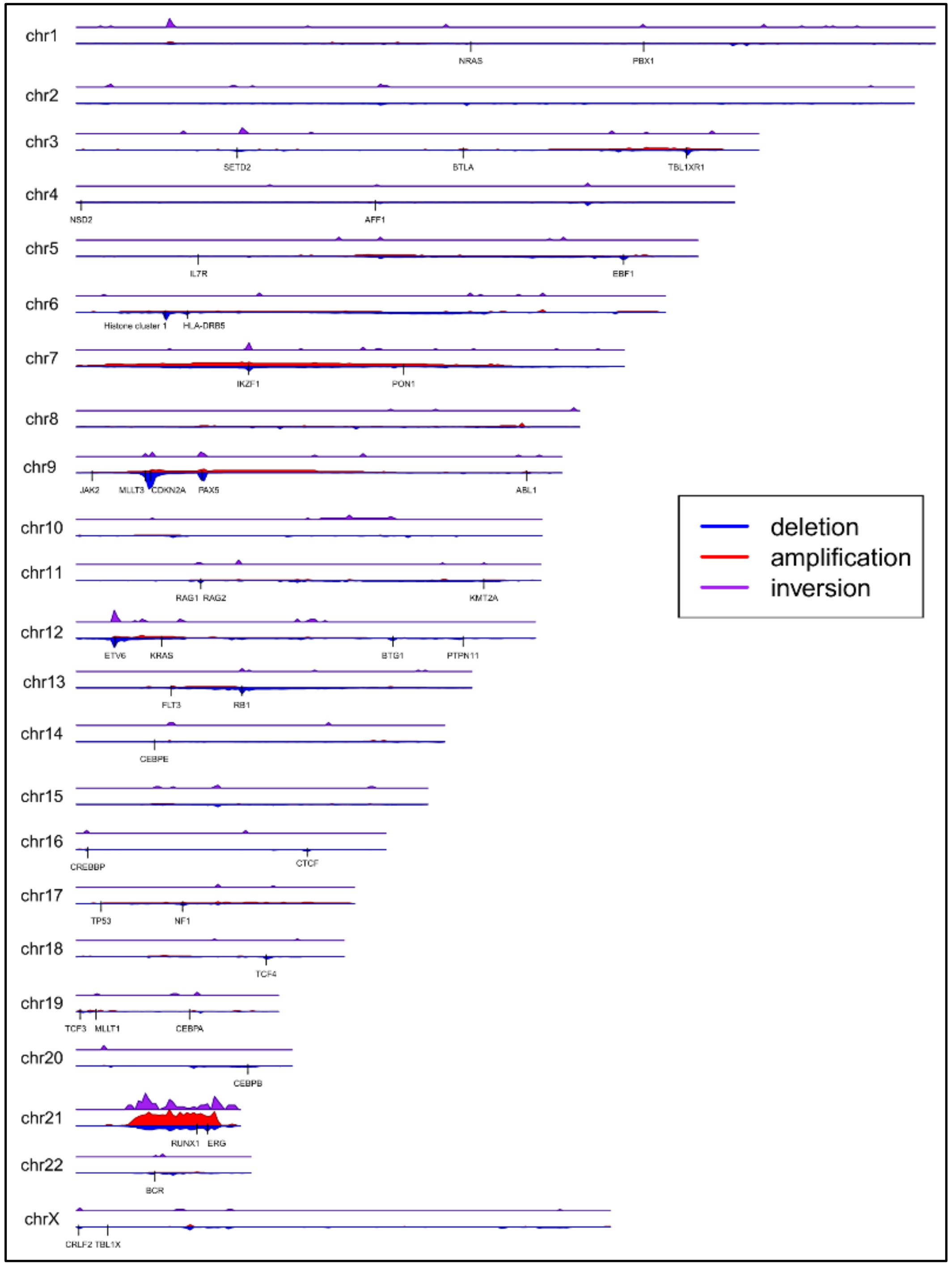

Supplementary Fig. 7

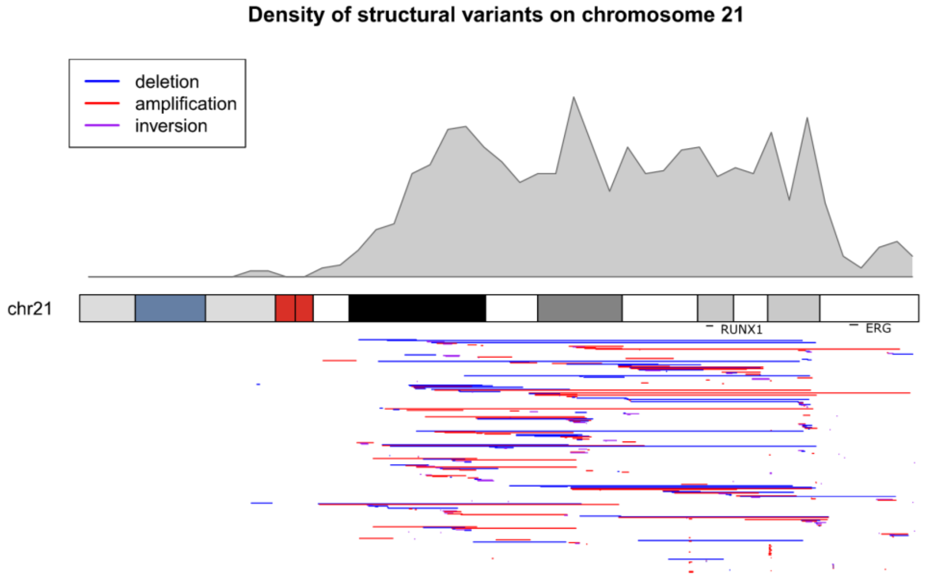

Supplementary Fig. 8

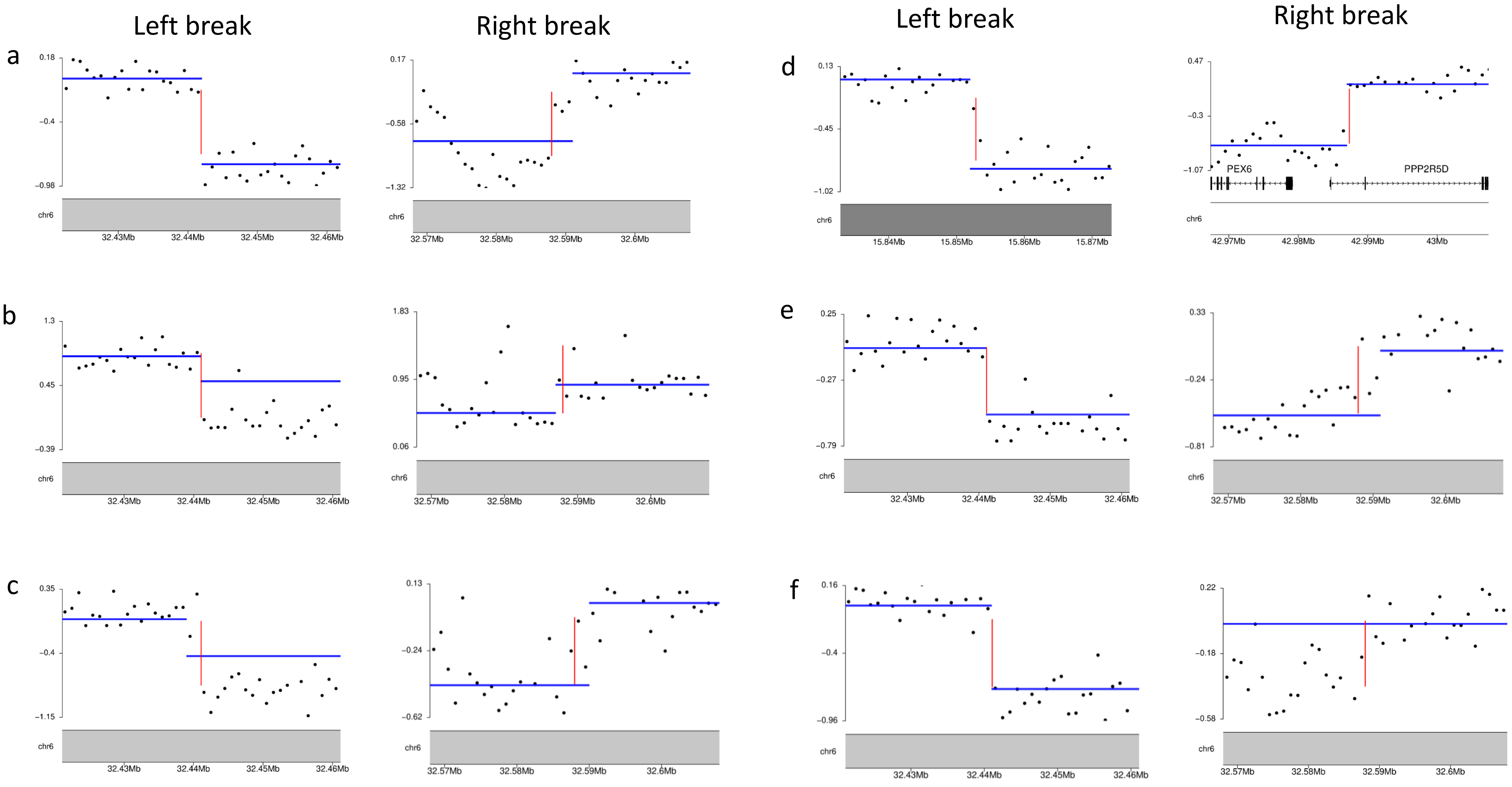

Supplementary Fig. 9

a

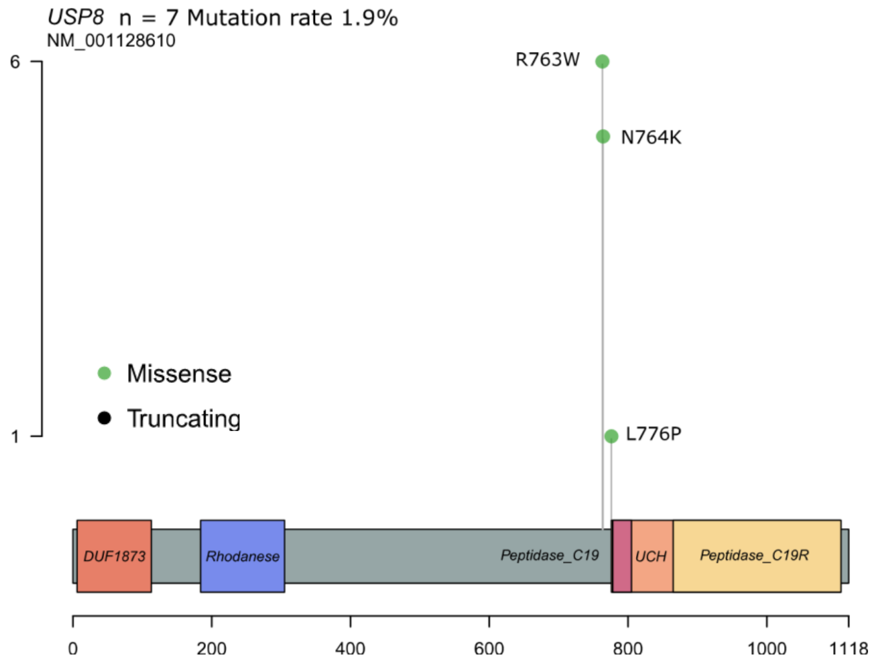

b

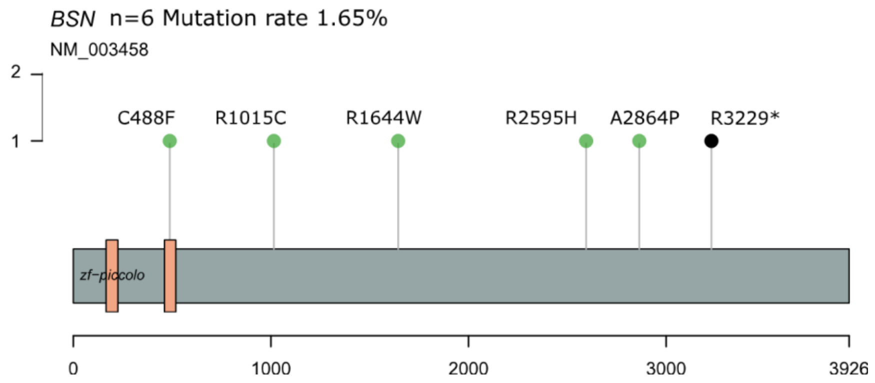

Supplementary Fig. 10

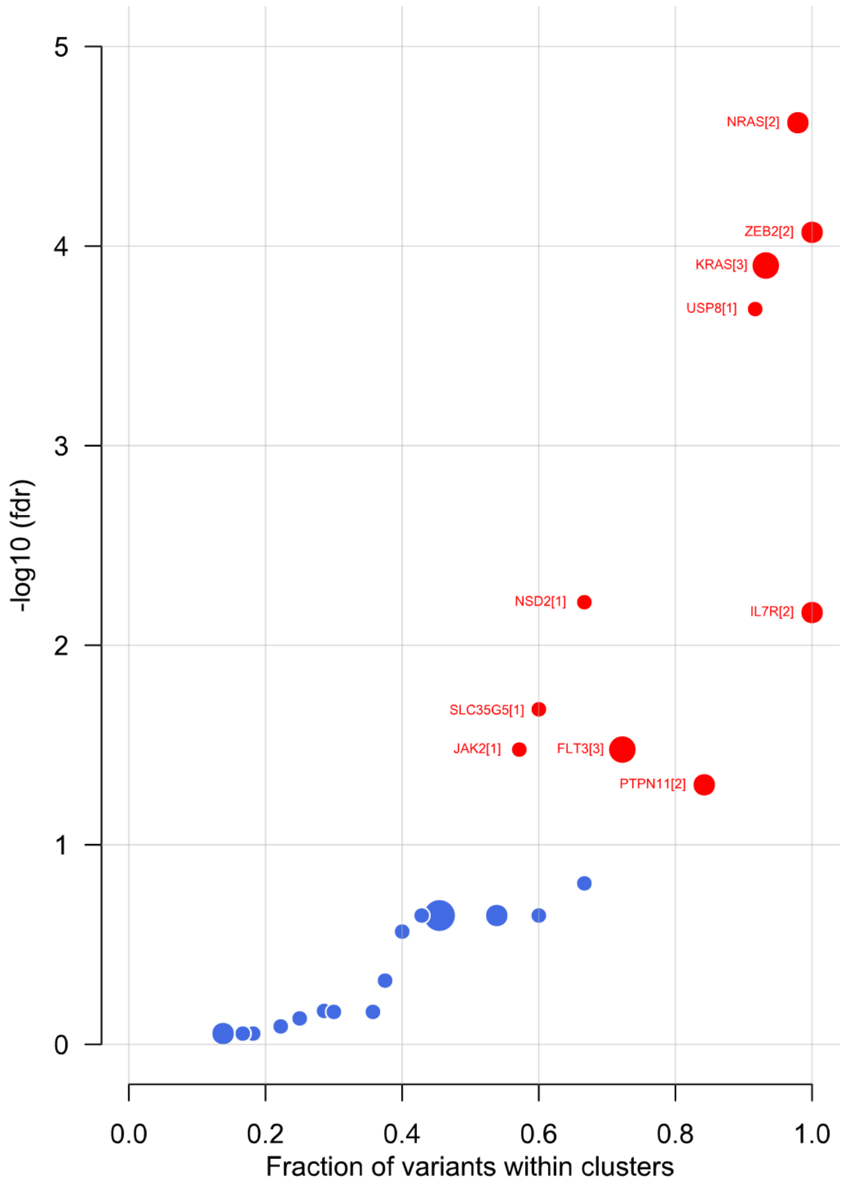

Supplementary Fig. 11

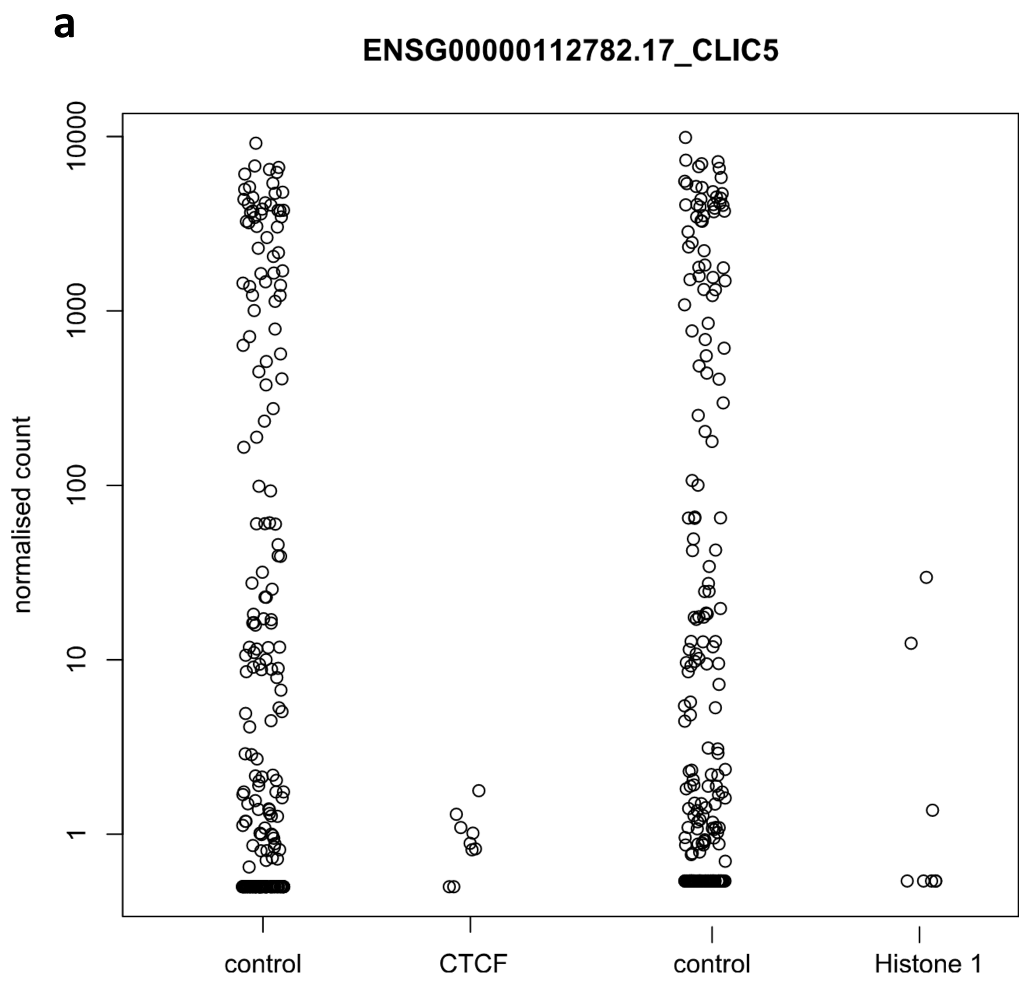

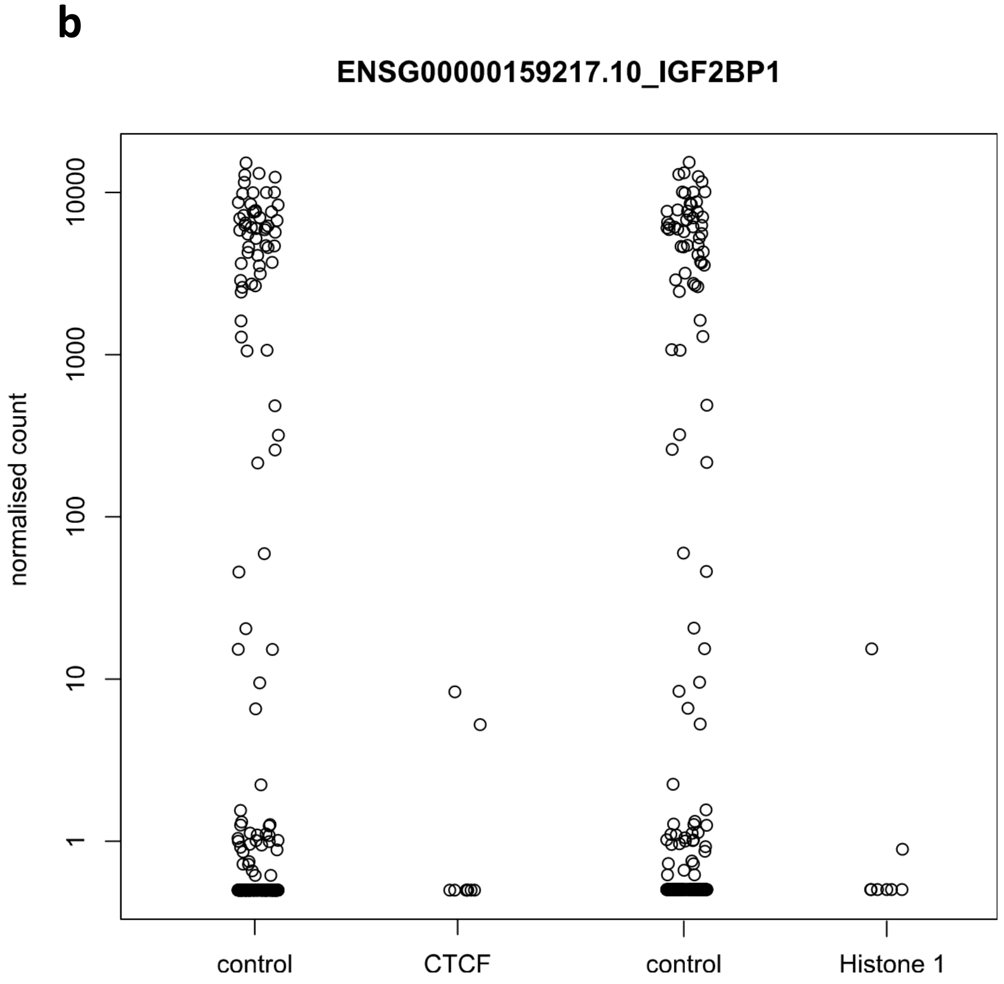

Supplementary Fig. 12

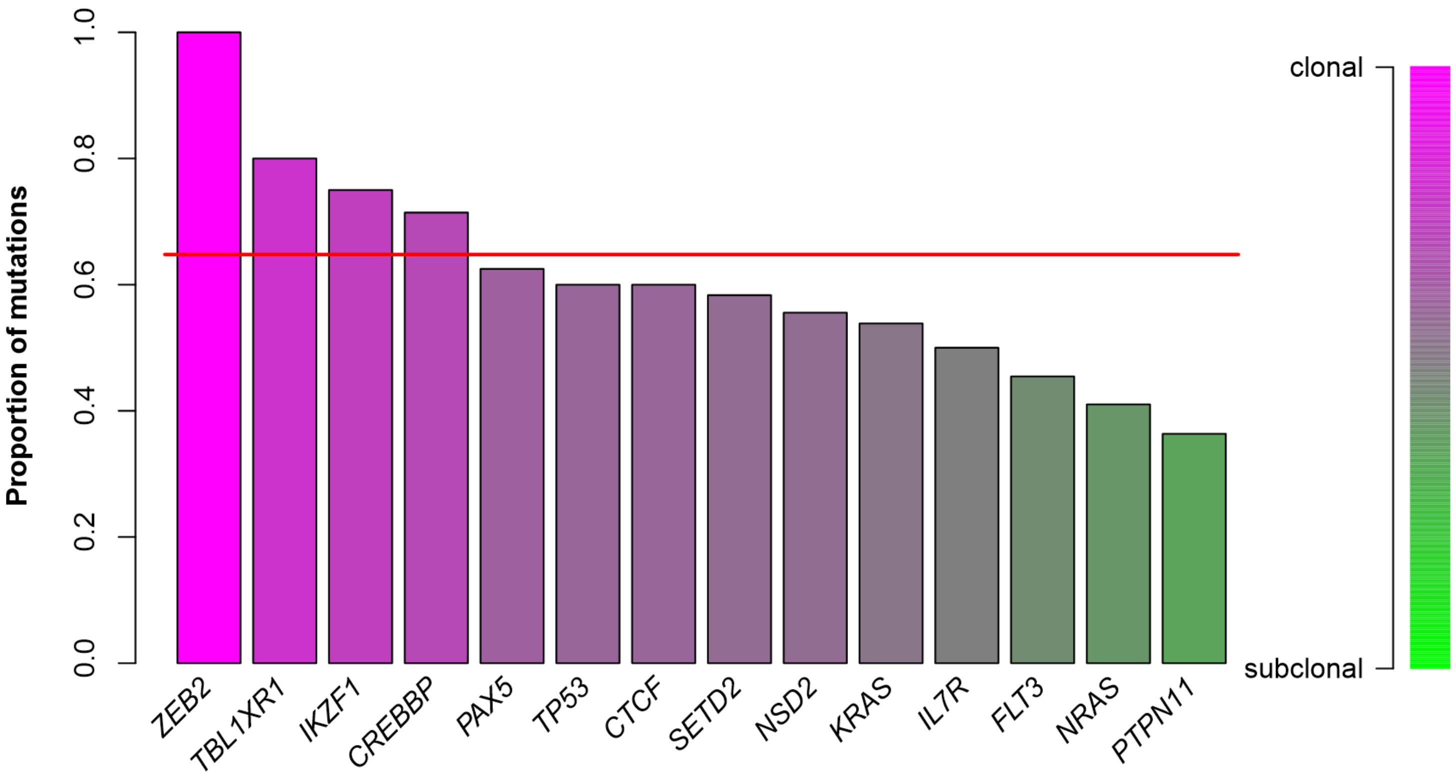

Supplementary Fig. 13

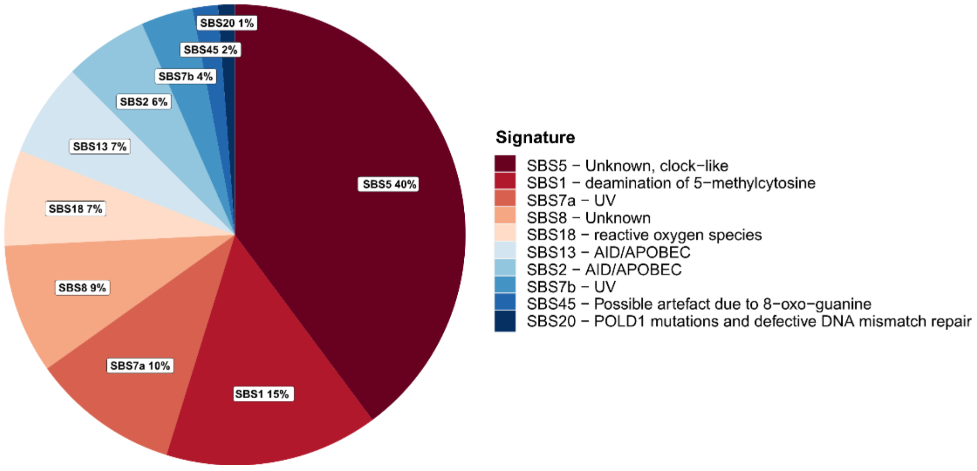

Supplementary Fig. 14

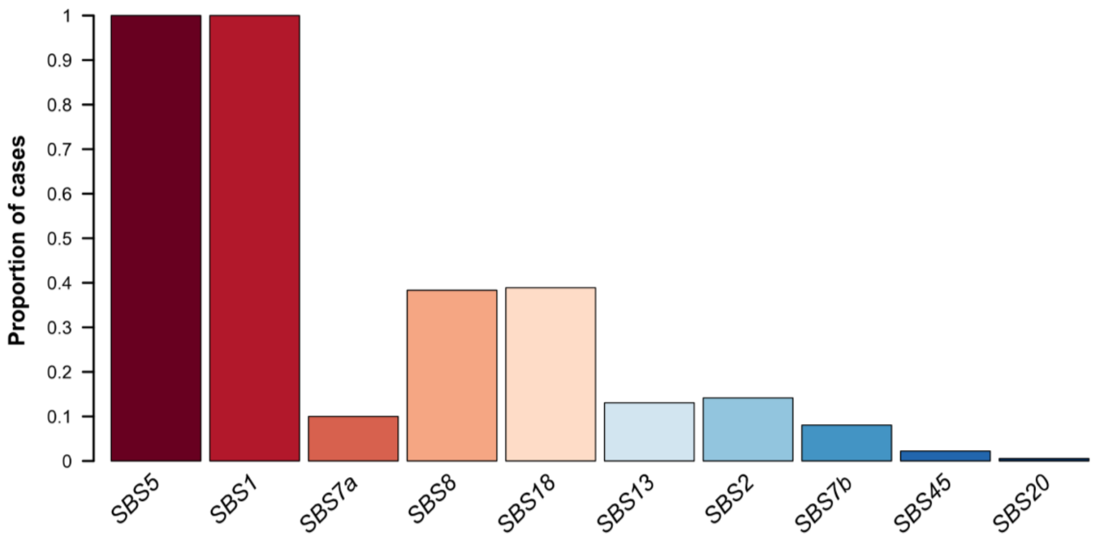

Supplementary Fig. 15

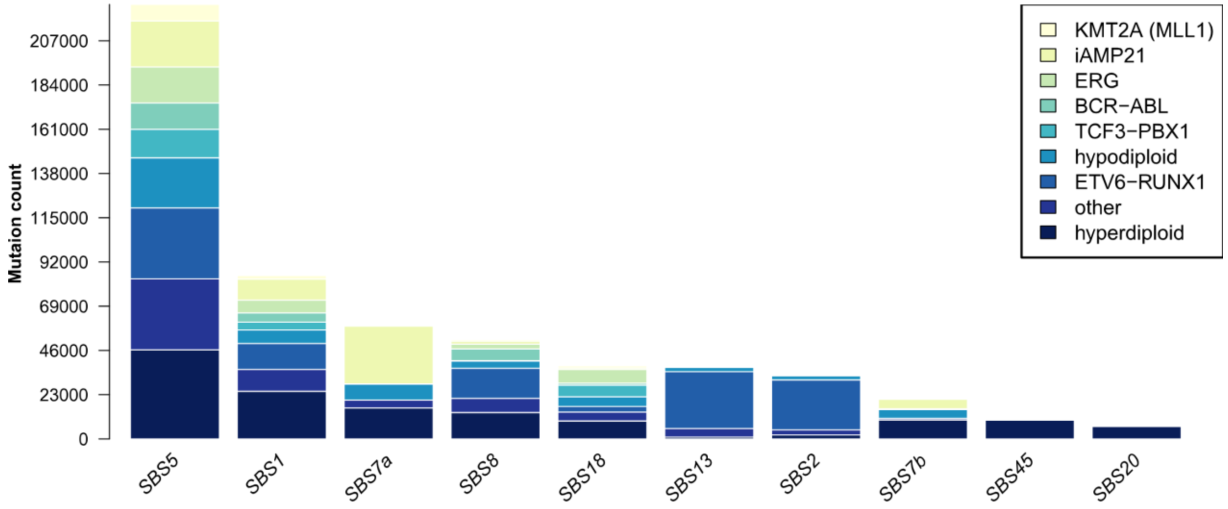

Supplementary Fig. 16

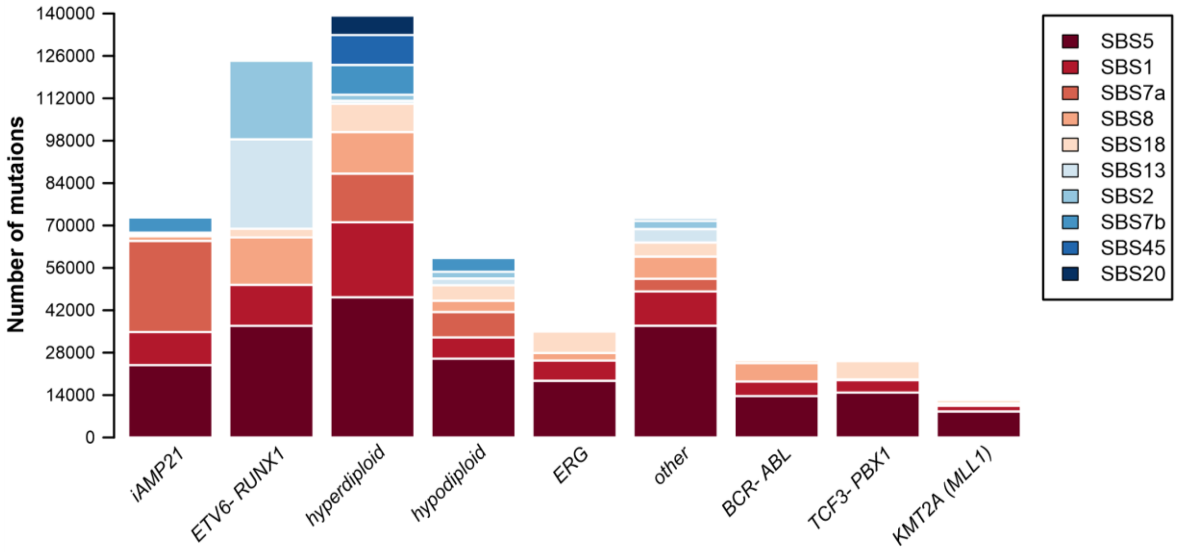

Supplementary Fig. 17

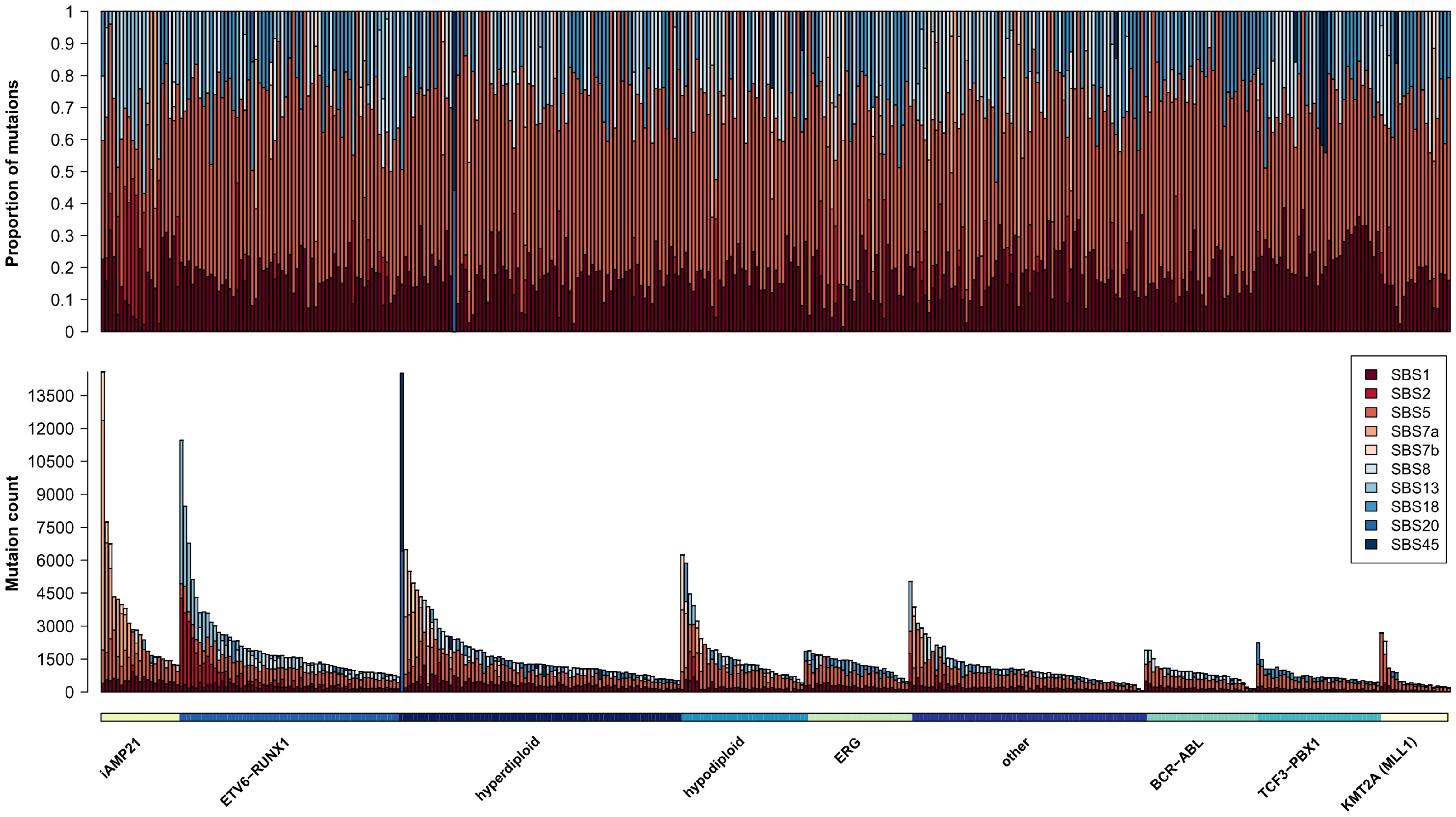

Supplementary Fig. 18

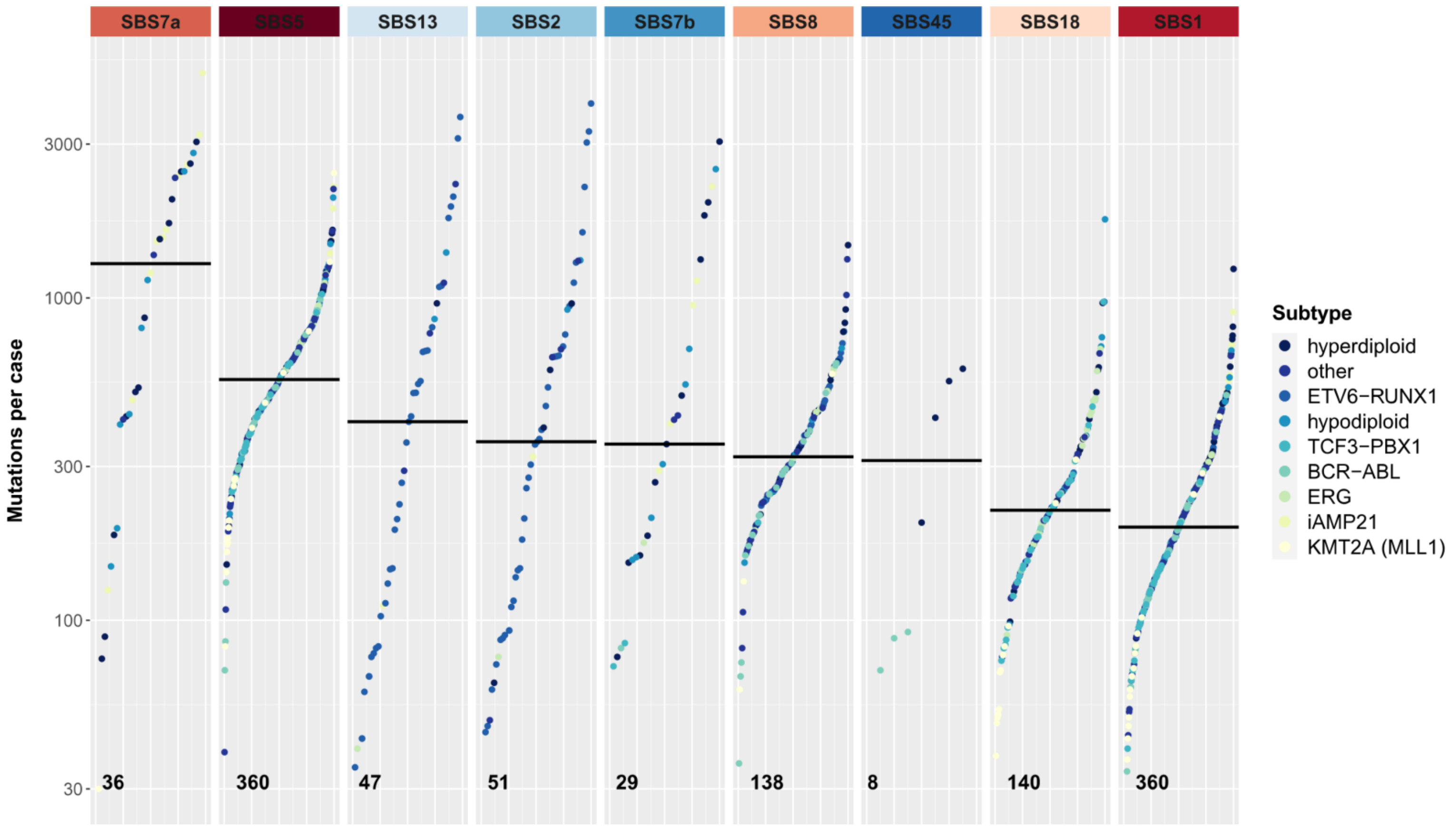

Supplementary Fig. 19

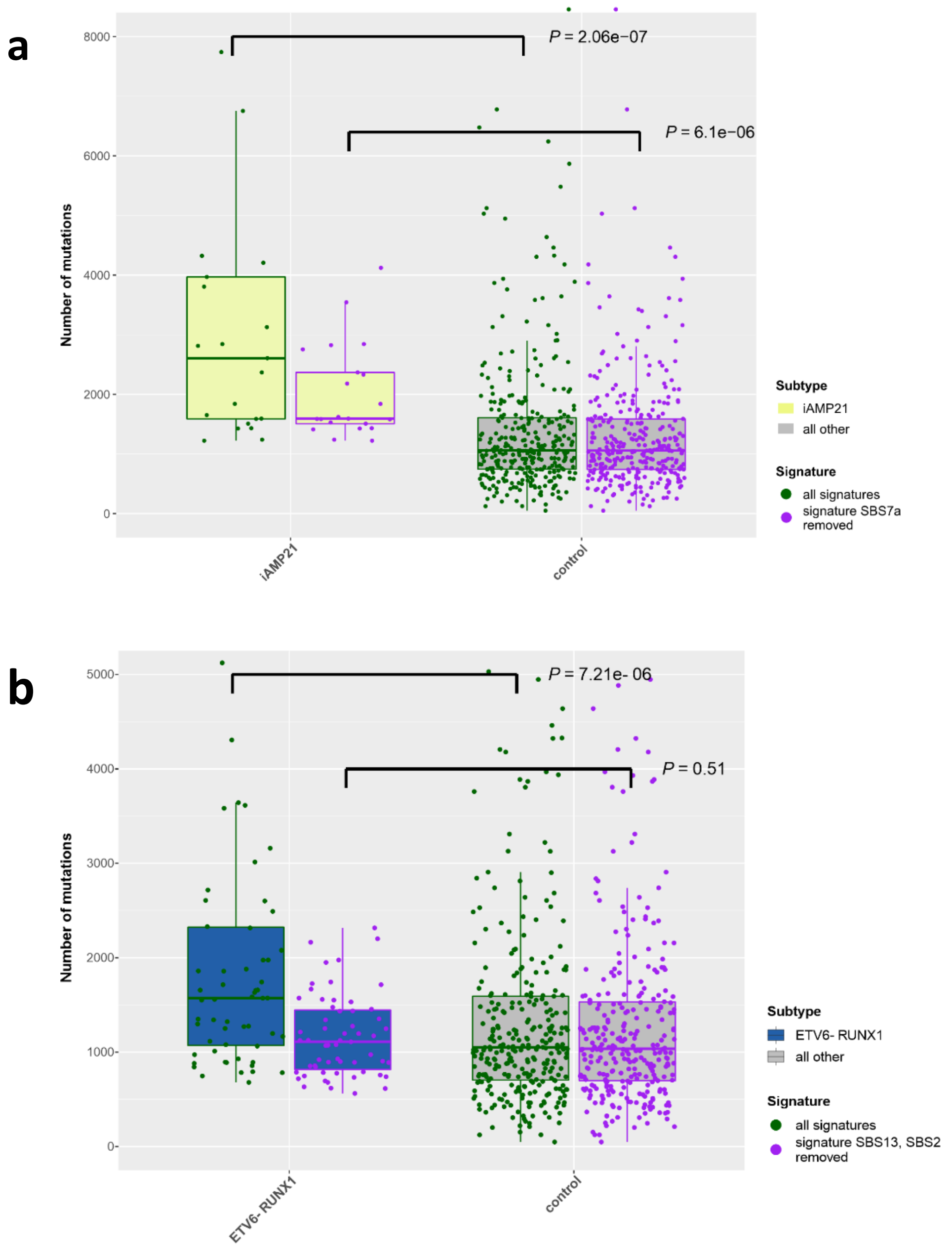

Supplementary Fig. 20

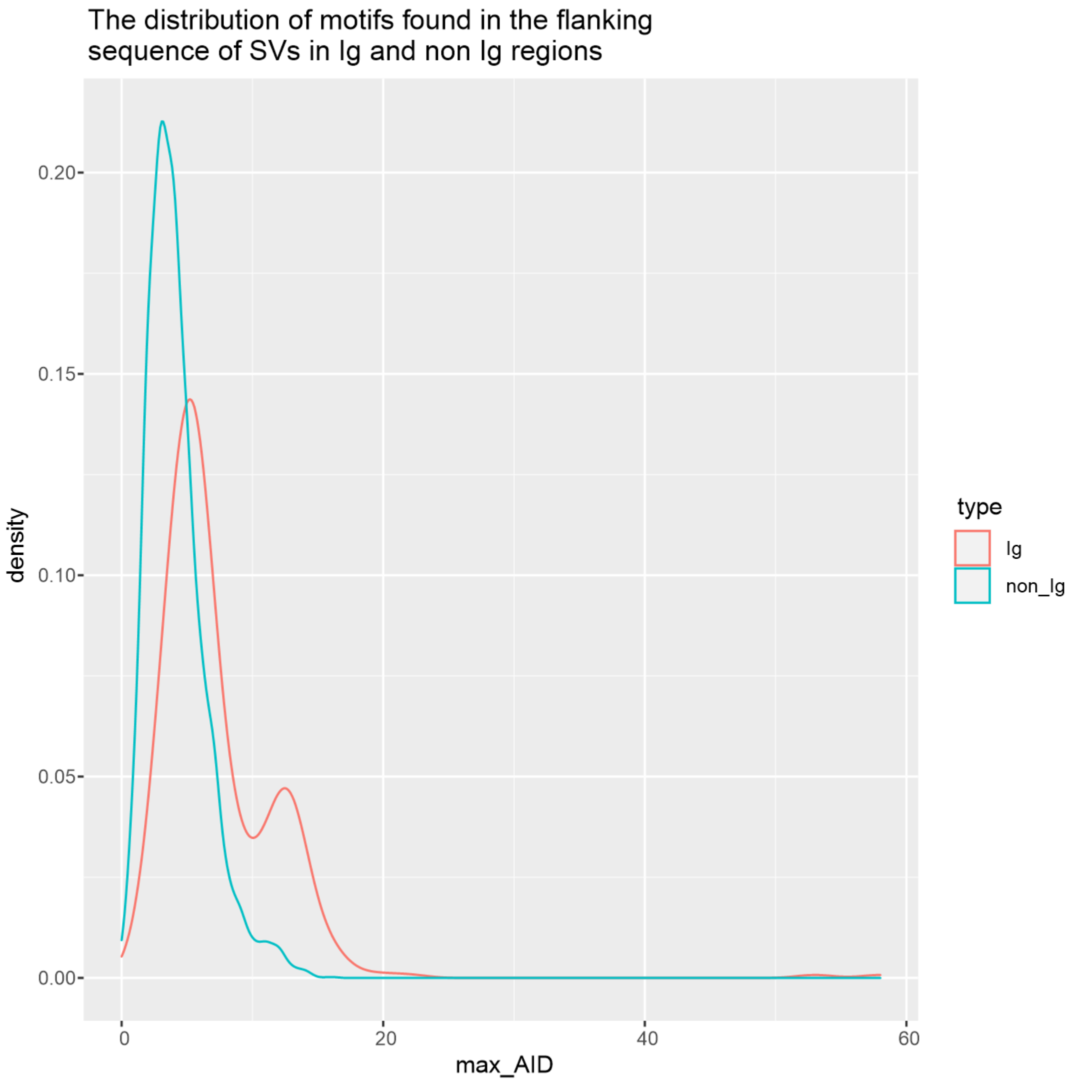

Supplementary Fig. 21

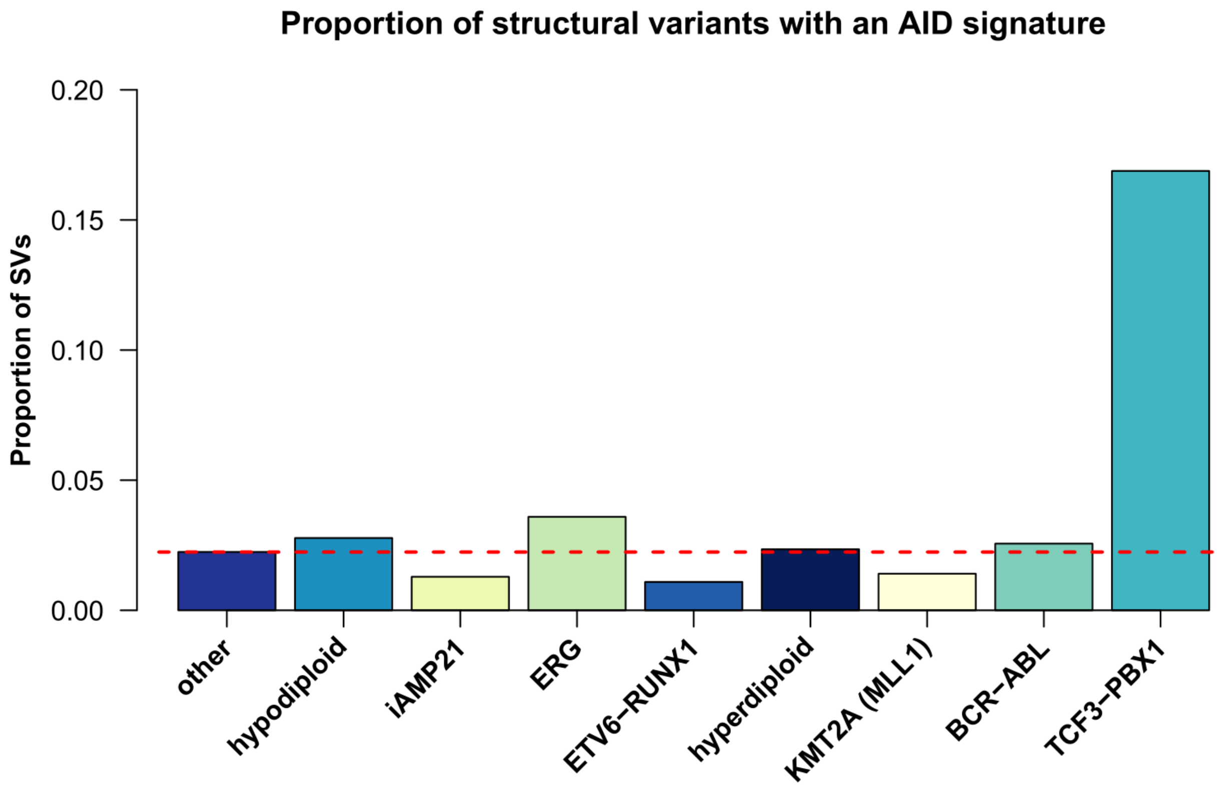

Supplementary Fig. 22

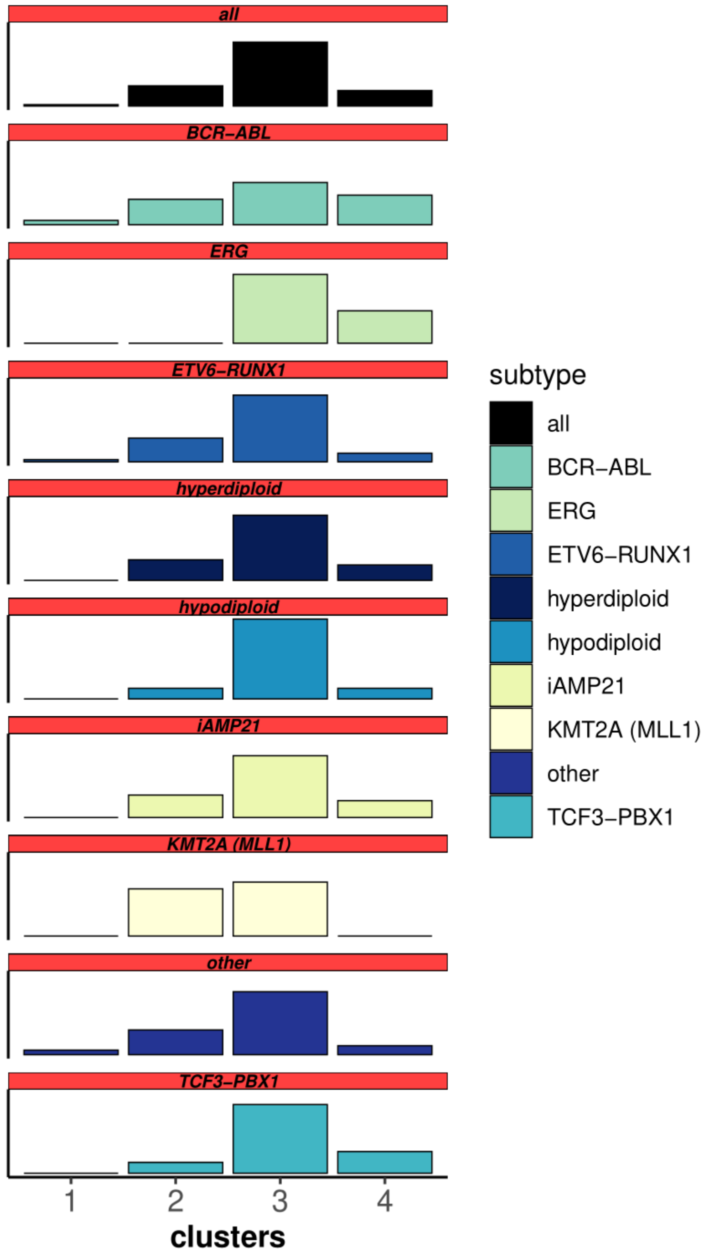

Supplementary Fig. 23

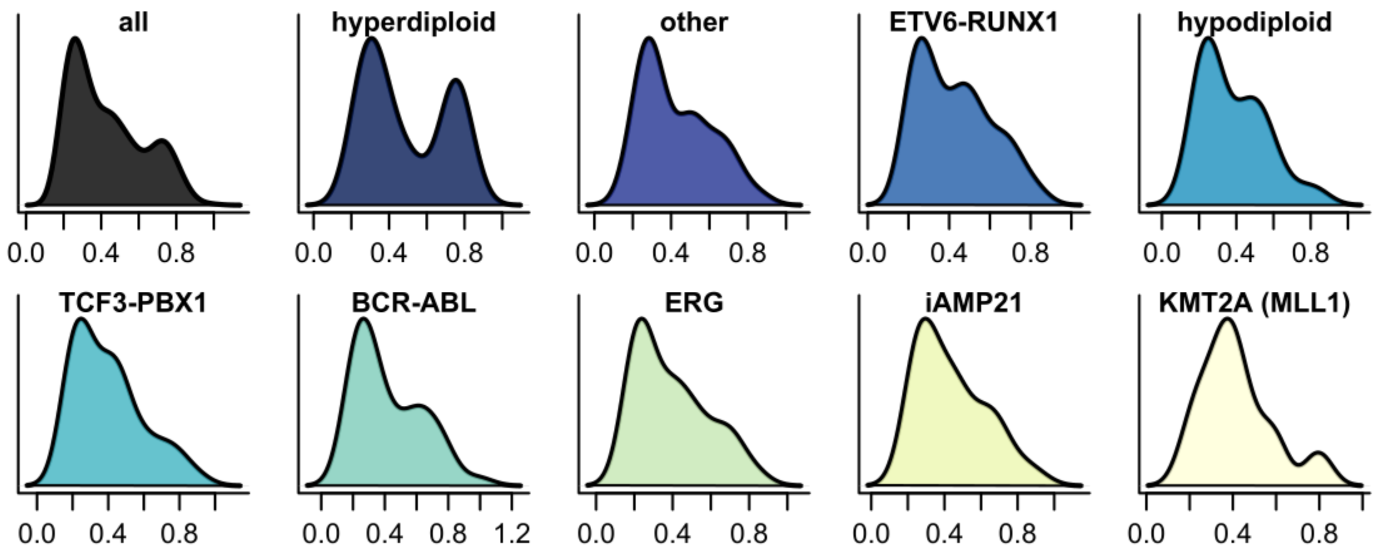

Supplementary Fig. 24

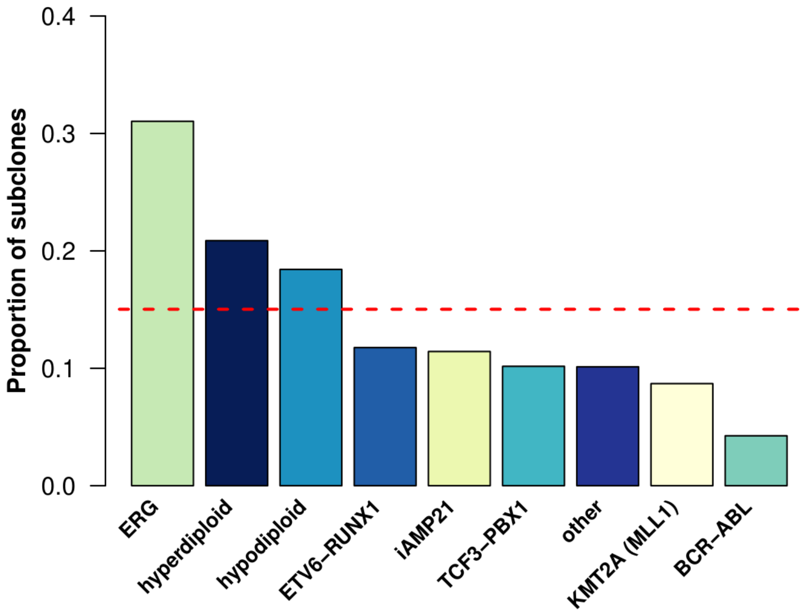

Supplementary Fig. 25

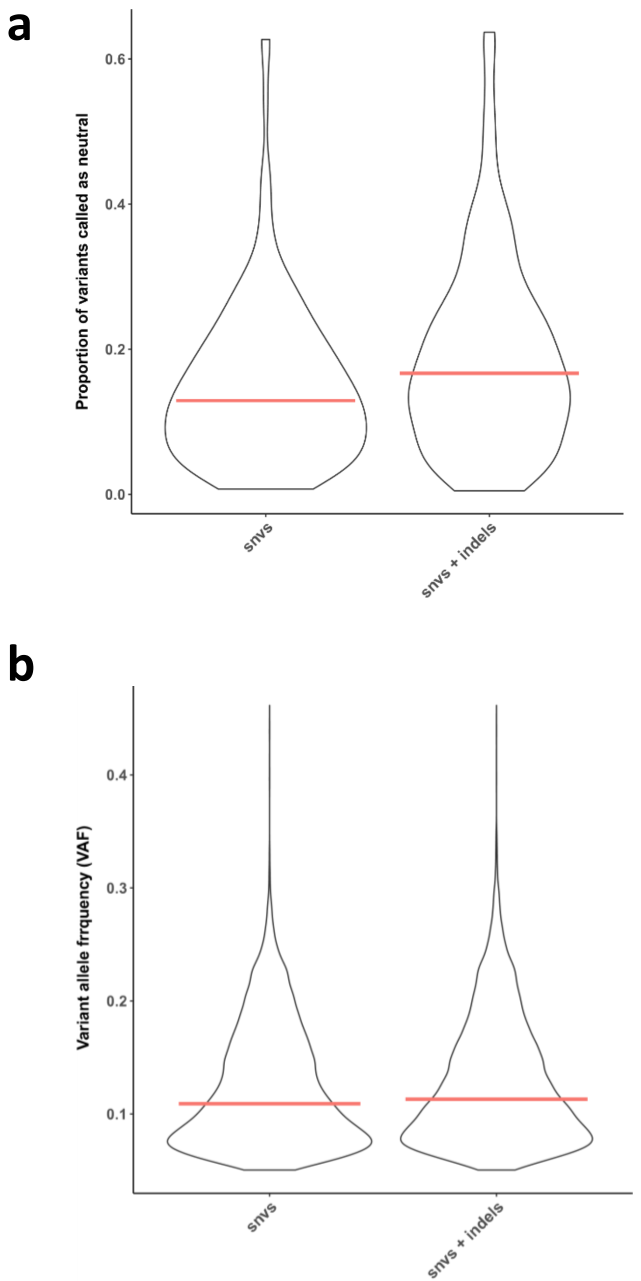

Supplementary Fig. 26

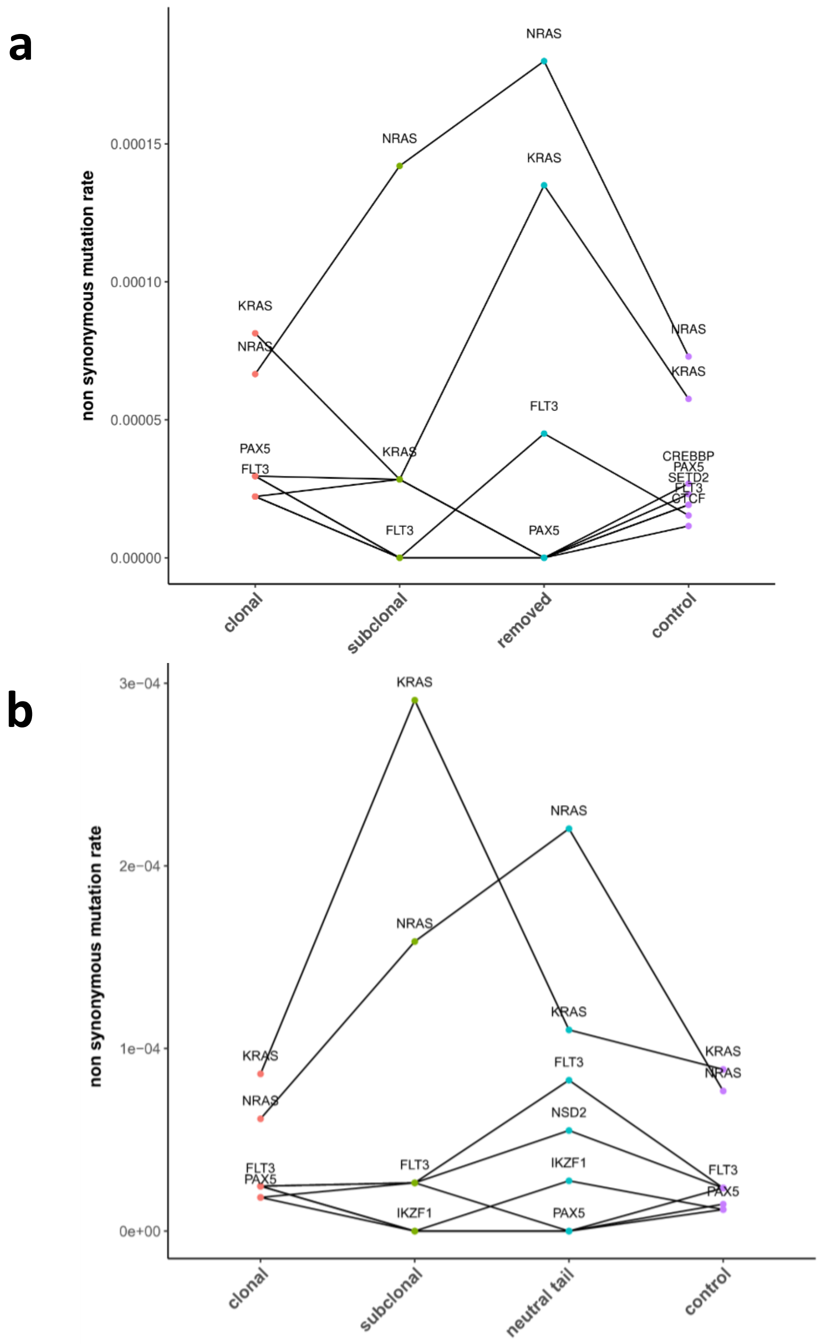

Supplement: Supplementary file 1 — Supplementary figures [file 41408_2021_570_MOESM1_ESM.pdf]
