# Supplementary material for: Cancer drivers and clonal dynamics in acute lymphoblastic leukaemia subtypes
Source: Blood Cancer J. 2021 Nov 9;11(11):177. doi: 10.1038/s41408-021-00570-9 (PMC8578656; doi:10.1038/s41408-021-00570-9)
Supplement: Supplementary file 2 — supplementary tables [file 41408_2021_570_MOESM2_ESM.docx]

**Supplementary table 1**

| **VEP damaging variants** |
| --- |
| missense_variant |
| protein_altering_variant |
| splice_acceptor_variant |
| splice_donor_variant |
| stop_gained |
| frameshift_variant |
| stop_lost |
| start_lost |
| inframe_insertion |
| inframe_deletion |
| transcript_amplification |
| transcript_ablation |

**Supplementary table 2**

| **Pathway** | **Gene** |
| --- | --- |
| RAS-RTK | *NRAS, KRAS, PTPN11, FLT3, NF1, ABL1* |
| B-cell development | *PAX5, IKZF1, ETV6, ZEB2, RUNX1, TCF3, RAG1, RAG2, EBF1* |
| Chromatin regulation | *SETD2, HDAC7, NSD2, CTCF, KMT2A, STAG2*, histone gene cluster 1 |
| Cytokine signalling | *JAK2, IL7R, CRLF2* |
| Signal transduction | *TBL1XR1, TBL1X, PBX1, PAG1* |
| Cell cycle regulation | *CDKN2A, CDKN2B, RB1* |
| Immune regulation | *BTLA, HLA-DRB5* |
| Gene regulation | *CREBBP, MLLT1, MLLT3, AFF1, BTG1, ERG, TCF4, NCOA6* |

**Supplementary table 3**

| **Chr** | **Start** | **Stop** | **Region** |
| --- | --- | --- | --- |
| Chr2 | 88857361 | 90235368 | IgK |
| chr7 | 142299011 | 142813287 | TRB |
| Chr7 | 38240024 | 38368055 | TRG |
| Chr14 | 21621904 | 22552132 | TRA |
| Chr14 | 105586437 | 106879844 | IgH |
| Chr22 | 22026076 | 22922913 | IgL |

**Supplementary table 4**

| **Motif name** | **Sequence** |
| --- | --- |
| E2A motif | CASSTG |
| PRDM9 motif | CCNCCNTNNCCNC |
| RAG heptamer | CACAGTG |
| RAG nonamer | ACAAAAACC |
| AID hotspot1 | GAGCT |
| AID hotspot2 | GGGST |
| AID hotspots3 | TGGGG |

**Supplementary table 5**

| **Gene** | **known / unknown** | **lesion type** |
| --- | --- | --- |
| NRAS | known | snv |
| KRAS | known | snv |
| PTPN11 | known | snv |
| FLT3 | known | snv |
| TP53 | known | snv |
| PAX5 | known | snv; copy |
| SETD2 | known | snv |
| IKZF1 | known | snv ; copy |
| CREBBP | known | snv |
| NSD2 | known | snv |
| TBL1XR1 | known | snv |
| ETV6 | known | snv ; copy ; translocation |
| NF1 | known | snv |
| IL7R | known | snv |
| CDKN2A | known | copy |
| TBL1X | known | snv |
| PON1 | known | snv |
| BTLA | known | copy;non_coding |
| CDKN2B | known | copy |
| RUNX1 | known | translocation |
| BCR | known | translocation |
| ABL1 | known | translocation |
| TCF3 | known | translocation ; copy |
| PBX1 | known | translocation ; copy |
| KMT2A | known | translocation |
| MLLT1 | known | translocation |
| MLLT3 | known | translocation |
| AFF1 | known | translocation |
| BTG1 | known | copy |
| RB1 | known | copy |
| ERG | known | copy |
| RAG1 | known | copy |
| RAG2 | known | copy |
| TCF4 | known | copy |
| EBF1 | known | copy |
| CRLF2 | known | translocation |
| CEBPA | known | translocation |
| CEBPB | known | translocation |
| CEBPE | known | translocation |
| CTCF | known | snv |
| ZEB2 | known | snv |
| JAK2 | known | snv |
| UBA2 | known | snv |
| CHID1 | unknown | copy;non_coding |
| HISTONE gene cluster 1 | unknown | copy |
| HLA-DRB5 | unknown | copy |
| USP8 | unknown | snv |
| SLC35G5 | unknown | snv |
| UBE2N | unknown | snv |
| BSN | unknown | snv |
| RBMX | unknown | snv |

**Supplementary table 6**

| **Gene** | **Mutation count** | **Tumour count** | **% mutated** | **Q value** |
| --- | --- | --- | --- | --- |
| KRAS | 59 | 56 | 15.5 | 6.43E-14 |
| NRAS | 49 | 46 | 12.7 | 6.43E-14 |
| CREBBP | 26 | 21 | 5.8 | 4.76E-13 |
| PTPN11 | 19 | 18 | 5.0 | 9.59E-11 |
| FLT3 | 25 | 18 | 5.0 | 5.23E-06 |
| SETD2 | 36 | 16 | 4.4 | 1.31E-04 |
| TP53 | 19 | 14 | 3.9 | 1.41E-09 |
| NSD2 | 12 | 12 | 3.3 | 1.19E-04 |
| PAX5 | 13 | 11 | 3.0 | 4.36E-06 |
| ETV6 | 13 | 10 | 2.8 | 3.22E-03 |
| KMT2D | 14 | 10 | 2.8 | 7.97E-03 |
| IL7R | 10 | 8 | 2.2 | 1.86E-07 |
| TBL1XR1 | 11 | 8 | 2.2 | 1.26E-03 |
| IKZF1 | 10 | 8 | 2.2 | 2.69E-03 |
| ZEB2 | 8 | 8 | 2.2 | 4.36E-03 |
| NF1 | 10 | 7 | 1.9 | 1.48E-03 |
| **USP8** | 12 | 7 | 1.9 | 3.22E-03 |
| JAK2 | 7 | 7 | 1.9 | 3.68E-02 |
| CTCF | 7 | 6 | 1.7 | 4.27E-04 |
| UBA2 | 8 | 6 | 1.7 | 2.74E-02 |
| **BSN** | 6 | 6 | 1.7 | 9.96E-02 |
| **SLC35G5** | 5 | 5 | 1.4 | 3.76E-03 |

**Supplementary table 7**

| **Gene** | **Chr** | **Position** | **Ref** | **Alt** | **dbSNP** | **Protein Change** | **SIFT** | **PolyPhen** | **count** | **Max VAF** | **Mean VAF** | **ExAC frequency** |
| --- | --- | --- | --- | --- | --- | --- | --- | --- | --- | --- | --- | --- |
| BSN | chr3 | 49643097 | G | T | novel | p.C488F | deleterious(0) | probably_damaging  (1) | 1 | 0.320755 | 0.320755 | 0 |
| BSN | chr3 | 49652599 | C | T | rs780669228 | p.R1015C | deleterious(0) | probably_damaging  (0.925) | 1 | 0.438356 | 0.438356 | 2.10e-4 |
| BSN | chr3 | 49654486 | C | T | rs747049829 | p.R1644W | deleterious(0) | probably_damaging  (0.999) | 1 | 0.486486 | 0.486486 | 0 |
| BSN | chr3 | 49657340 | G | A | rs760395148 | p.R2595H | deleterious(0) | probably_damaging  (0.972) | 1 | 0.407895 | 0.407895 | 8.33e-6 |
| BSN | chr3 | 49658146 | G | C | novel | p.A2864P | deleterious(0.01) | Benign  (0.38) | 1 | 0.068966 | 0.068966 | 0 |
| BSN | chr3 | 49661530 | C | T | rs1275038095 | p.R3229* |  |  | 1 | 0.212121 | 0.212121 | 0 |
| USP8 | chr15 | 50492753 | C | T | rs78143971 | p.R763W | deleterious(0.02) | probably_damaging  (0.958) | 6 | 0.135802 | 0.100436 | 0.34 |
| USP8 | chr15 | 50492758 | C | A | rs74840283 | p.N764K | deleterious(0.01) | Benign  (0.046) | 5 | 0.191011 | 0.120657 | 0.34 |
| USP8 | chr15 | 50492793 | T | C | rs146125856 | p.L776P | deleterious(0) | probably_damaging  (0.968) | 1 | 0.149254 | 0.149254 | 8.42e-3 |
| SLC35G5 | chr8 | 11331297 | G | C | rs753517750 | p.G64A | tolerated(0.73) | benign(0) | 1 | 0.142857 | 0.142857 | 9.06e-5 |
| SLC35G5 | chr8 | 11331413 | T | C | rs76944947 | p.W103R | tolerated(1) | benign(0) | 1 | 0.333333 | 0.333333 | 2.34e-2 |
| SLC35G5 | chr8 | 11331675 | C | T | rs115295428 | p.T190I | tolerated(0.35) | benign(0) | 3 | 0.184211 | 0.144418 | 7.33e-2 |

**Supplementary table 8**

| **Transcription factor** | **Count** | **Frequency** |
| --- | --- | --- |
| RUNX1 | 15 | 1 |
| GATA3 | 15 | 1 |
| MYB | 15 | 1 |
| RUNX3 | 15 | 1 |
| TBX21 | 15 | 1 |
| BCL6 | 15 | 1 |
| NFIC | 15 | 1 |
| SPI1 | 14 | 0.93 |
| MEF2B | 14 | 0.93 |
| STAT4 | 14 | 0.93 |
| GABPA | 4 | 0.27 |
| MAX | 7 | 0.47 |
| TAL1 | 7 | 0.47 |
| ATF2 | 3 | 0.2 |

**Supplementary table 9**

| **Gene** | **Subtype** | **Lesion** | **Subtype count** | **Subtype percent** | **Control count** | **Control percent** | **Fold enrichment** | **P value** | **Q value** |
| --- | --- | --- | --- | --- | --- | --- | --- | --- | --- |
| NF1 | hypodiploid | SNV | 6 | 18.2 | 1 | 0.3 | 59.6 | 2.5E-06 | 3.8E-04 |
| TBL1XR1 | ERG | SNV | 6 | 21.4 | 2 | 0.6 | 35.7 | 3.2E-06 | 4.9E-04 |
| IL7R | iAMP21 | SNV | 4 | 19.0 | 4 | 1.2 | 16.2 | 5.2E-04 | 7.9E-02 |
| FLT3 | hyperdiploid | SNV | 14 | 18.7 | 4 | 1.4 | 13.3 | 1.5E-07 | 2.3E-05 |
| ZEB2 | ERG | SNV | 4 | 14.3 | 4 | 1.2 | 11.9 | 1.7E-03 | 8.4E-02 |
| PBX1 | TCF3-PBX1 | Amp | 13 | 43.3 | 16 | 5.3 | 8.1 | 5.1E-08 | 1.0E-05 |
| CREBBP | hyperdiploid | SNV | 14 | 18.7 | 7 | 2.4 | 7.6 | 3.3E-06 | 1.7E-04 |
| TP53 | hypodiploid | SNV | 6 | 18.2 | 8 | 2.4 | 7.5 | 6.6E-04 | 5.0E-02 |
| KRAS | ETV6-RUNX1 | Del | 8 | 13.6 | 6 | 2.2 | 6.1 | 8.4E-04 | 9.5E-02 |
| RB1 | iAMP21 | Del | 8 | 40.0 | 21 | 6.8 | 5.9 | 8.2E-05 | 2.8E-02 |
| TCF3 | TCF3-PBX1 | Del | 10 | 33.3 | 21 | 7.0 | 4.8 | 1.0E-04 | 3.5E-02 |
| PAG1 | ETV6-RUNX1 | Del | 7 | 11.9 | 7 | 2.6 | 4.6 | 5.1E-03 | 4.4E-01 |
| TBL1XR1 | ETV6-RUNX1 | Del | 13 | 22.0 | 13 | 4.8 | 4.6 | 9.0E-05 | 1.5E-02 |
| ETV6 | ETV6-RUNX1 | Del | 38 | 64.4 | 42 | 15.5 | 4.2 | 1.8E-13 | 6.1E-11 |
| KRAS | hyperdiploid | SNV | 26 | 34.7 | 30 | 10.5 | 3.3 | 2.5E-06 | 1.7E-04 |
| NRAS | ERG | SNV | 10 | 35.7 | 36 | 10.8 | 3.3 | 9.8E-04 | 7.5E-02 |

**Supplementary table 10**

|  | **histone 1** | | | | **CTCF** | | | |
| --- | --- | --- | --- | --- | --- | --- | --- | --- |
| gene name | fold Change (log2) | p value | q value | rank | fold Change (log2) | p value | q value | rank |
| NOVA1 | 22.17 | 1.95E-22 | 8.57E-19 | 4 | 9.64 | 7.89E-06 | 0.012334 | 14 |
| DSC3 | 26.42 | 2.14E-20 | 6.28E-17 | 6 | 27.87 | 7.84E-25 | 5.31E-21 | 3 |
| CLIC5 | 7.16 | 5.68E-06 | 4.68E-03 | 22 | 10.63 | 9.06E-12 | 4.6E-08 | 4 |
| IGF2BP1 | 9.57 | 5.10E-05 | 0.028 | 32 | 9.89 | 9.00E-06 | 0.013067 | 15 |
| KCNA2 | 6.72 | 6.36E-05 | 0.032 | 36 | 5.79 | 1.62E-04 | 0.070069 | 47 |

**Supplementary table 11**

|  | All | hyperdiploid | other | ETV6-RUNX1 | hypodiploid | TCF3-PBX1 | BCR-ABL | ERG | iAMP21 | KMT2A_MLL1 |
| --- | --- | --- | --- | --- | --- | --- | --- | --- | --- | --- |
| RAS/RTK | 0.51 | 0.76 | 0.49 | 0.46 | 0.53 | 0.33 | 0.07 | 0.61 | 0.57 | 0.47 |
| B-cell development | 0.68 | 0.51 | 0.70 | 0.86 | 0.65 | 0.70 | 0.73 | 0.75 | 0.95 | 0.26 |
| Chromatin regulation | 0.34 | 0.30 | 0.32 | 0.56 | 0.29 | 0.18 | 0.20 | 0.36 | 0.57 | 0.11 |
| Cytokine signalling | 0.10 | 0.18 | 0.16 | 0.07 | 0.06 | 0.00 | 0.00 | 0.00 | 0.38 | 0.00 |
| Gene regulation | 0.59 | 0.57 | 0.52 | 0.59 | 0.82 | 0.48 | 0.43 | 0.71 | 0.95 | 0.32 |
| Signal Transduction | 0.27 | 0.20 | 0.16 | 0.42 | 0.32 | 0.48 | 0.07 | 0.36 | 0.38 | 0.11 |
| Cell cycle regulation | 0.38 | 0.28 | 0.37 | 0.36 | 0.50 | 0.42 | 0.57 | 0.36 | 0.52 | 0.16 |
| Immune Regulation | 0.09 | 0.03 | 0.14 | 0.12 | 0.03 | 0.00 | 0.10 | 0.11 | 0.29 | 0.00 |
