# Supplementary material for: Cancer drivers and clonal dynamics in acute lymphoblastic leukaemia subtypes
Source: Blood Cancer J. 2021 Nov 9;11(11):177. doi: 10.1038/s41408-021-00570-9 (PMC8578656; doi:10.1038/s41408-021-00570-9)
Supplement: Supplementary file 3 — SUPPLEMENTARY FIGURE and TABLE LEGENDS [file 41408_2021_570_MOESM3_ESM.docx]

**SUPPLEMENTARY FIGURES LEGENDS**

**Supplementary Figure 1**. Study overview.

**Supplementary Figure 2**. Molecular subtype composition. Due to small numbers near haploid (chromosome copy number 23-30) tumours were grouped with hypodiploid tumours and *BCR-ABL* like with unclassified/other.

**Supplementary Figure 3**. Frequency distribution of tumour purity as assessed by Battenberg, number of tumours=280.

**Supplementary Figure 4**. Chromosomal arm aberrations. Stacked bar charts of alteration frequency. Left Y-axis - percent of tumours. Right Y-axis number of tumours.

**Supplementary Figure 5**. Frequency of large-scale chromosomal aberrations, by ALL subtype. (**a**) 9p deletion; (**b**) 21q amplification.

**Supplementary Figure 6.** Genome-wide density plots of structural variants (excluding interchromosomal and immunoglobin loci events). Inversions, upper track, displayed in purple, amplifications, lower track, displayed in red and deletions, lower track, displayed in blue. Density calculated over the interval between SV breakpoints and shown on a genome-wide scale.

**Supplementary Figure 7**. Density and regional genetic plot of structural variant (SV) distribution across chromosome 21. Ideogram cytobands based on GRCh38 coordinates. The location of *RUNX1* and *ERG* are shown for reference. Lower horizontal line depict individual variants, blue – deletions; red – amplifications; purple – inversions.

**Supplementary Figure 8**. Copy number changes around structural variant breakpoints overlapping *HLA-DRB5*. Tumours with structural variants overlapping chr6-32,442,465-32,554,750 but lacking supporting CNVs were analysed using an additional CNV caller (HMMcopy). Dots show the normalised (mappability and GC) log2 tumour/normal coverage ratio for 1000bp bins. Horizontal blue lines show copy number segments called by HMMcopy and vertical red line show the location of SV breakpoints. All coordinates from GRCh38.

**Supplementary Figure 9**. Mutation plots of (**A**) *ZEB2,* (**B**) *CTCF* and (**C**) *MAP1B*. Lollipops represent the position of mutations within the protein. Lollipop height indicates mutation count and text shows the specific amino acid substitutions, plot produced using Maftools^6^.

**Supplementary Figure 10**. Mutations in *ZEB2*, *IL7R* and *JAK2* cluster into hotspots, consistent with oncogenic activation. OncodriveCLUST plot, x axis shows the frequency of non-silent variants located with clusters. Y axis –log_10_FDR corrected *P*-value.

**Supplementary Figure 11**. Expression of genes in *CTCF* and histone gene cluster 1 altered tumours. Normalised RNAseq counts of *CLIC5* (**a**) and *IGF2BP1* (**b**) extracted by DESeq2.

**Supplementary Figure 12**. Driver gene mutation (SNV/indels) clonality. Proportion of non-silent variants assigned to clonal clusters. Horizontal red line depicts the proportion of clonal mutations of all driver genes.

**Supplementary Figure 13.** Proportion of mutations attributed to COSMIC single base mutation signatures (v3), extracted using SigProfilerExtractor^29^.

**Supplementary Figure 14**. Prevalence of mutational signatures. Y-axis, the proportion of tumours in which COSMIC mutation signatures observed.

**Supplementary Figure 15.** Subtype mutation count, split by COSMIC signature.

**Supplementary Figure 16.** Mutation count attributed to each COSMIC signature, split by tumour subtype.

**Supplementary Figure 17**. Mutation signature profile in 361 tumours. Each bar represents a tumour. Upper pane shows the proportion of mutations assigned to signatures. Lower pane shows the mutation count per signature. Lower multi-coloured horizontal bar denotes tumour subtype.

**Supplementary Figure 18**. COSMIC signature mutation burden and frequency distribution. Solid horizontal black line denotes the median mutation count per tumour. Y-axis; mutation count. X-axis; cumulative frequency, 0-100% from left to right. Each dot represents a tumour and colour denotes subtype. The lower number in bold - number of tumours with signature mutations.

**Supplementary Figure 19.** Mutational signatures SBS7a and SBS13/SBS2 drive higher mutations rates in iAMP21 and *ETV6-RUNX1* tumours respectively. Box and whiskers plot of mutation count. Each dot denotes a tumour. Purple outline denotes mutations per tumour after remove variants attributed to (**a**) SBS7a (iAMP21) and (**b**) SBS13/SBS2 (*ETV6-RUNX1*). Control group contains all tumours after the removal of (**a**) iAMP21 and (**b**) *ETV6-RUNX1*.

**Supplementary Figure 20**. The number of repeats of core *AID* motifs in structural variant (SV) breakpoints in immunoglobulin regions and non Ig loci. 500bp sequences flanking SV breakpoints was extracted and the number of AID core motifs enumerated. Y-axis density and X-axis motif number per SV.

**Supplementary Figure 21**. The proportion of SVs with an AID motif count >10. Red horizontal red line shows the average for all tumours.

**Supplementary Figure 22**. Subclonal reconstruction showing the number of clones in tumour subtypes. Bar chart of cluster counts (clonal and subclonal) in each tumour. Y-axis; proportion of tumours. X-axis; number of clusters.

**Supplementary Figure 23**. Subclone frequency distribution. Density plot of subclone cancer cell fraction (CCF). For each subtype the subclone CCF is plotted. Y-axis; density. X-axis; subclone CFF.

**Supplementary Figure 24**. Proportion of subclones with a driver mutation based on **Supplementary table 10**.

**Supplementary Figure 25**. Violin plots of variants fitted as neutral by MOBSTER^53^; (**a**) proportion of variants fitted per tumour (**b**) VAF of fitted variants.

**Supplementary Figure 26**. Non-synonymous mutation rates of ALL drivers in clonal, subclonal and neutral compartments. Line graph shows the rate of nonsynonymous mutations. Variants; (**a**) SNVs and (**b**) SNVs and indels consistent with neutral evolution were identified using MOBSTER^53^.

**SUPPLEMENTARY TABLE LEGENDS**

**Supplementary Table 1**. Non-silent/damaging variant effect predictor annotations.

**Supplementary Table 2**. Biological pathway gene assignment used for pathway analysis.

**Supplementary Table 3**. Immunoglobulin regions used for filtering somatic variants. Coordinates from GRCh38.

**Supplementary Table 4**. Motifs of candidate mutagenic drivers in tumours, used for the motif enrichment in SV breakpoint analysis.

**Supplementary Table 5.** Previously reported ALL driver genes.

**Supplementary Table 6**. Driver genes identified from short variant analysis. Table compiled from the output of dndsCV, MutSigCV2 and OncodriveFML using SNVs and indels retaining only genes with significant *P*-values in ≥2. *Q*-values calculated using FDR correction (Benjamini-Hochberg). Undescribed genes in bold.

**Supplementary Table 7**. Non-silent variants found in candidate novel driver genes.

**Supplementary Table 8.** Disrupted transcription motifs in the promoter of *BTLA*. 15 tumours possessed *BTLA*-promoter mutations which were predicted to disrupt the binding of overlapping transcription factors. Table shows number of tumours with a mutation predicted to disrupt the motif of a binding transcription.

**Supplementary Table 9.** Frequency of genetic alteration in tumour subtypes, excluding subtype-initiating lesions.

**Supplementary Table 10.** Differentially expressed genes in histone gene cluster 1 deleted and *CTCF* altered tumours identified by DESeq2. Rank refers to the *P*-value ranked position of that gene in the list of most significantly differentially expressed genes within each data set.

**Supplementary Table 11**. Frequency of alterations to gene pathways. Gene pathway assignments based on **Supplementary table 2.**
